# Cervicovaginal Immune Activation in Zambian Women With Female Genital Schistosomiasis

**DOI:** 10.3389/fimmu.2021.620657

**Published:** 2021-03-02

**Authors:** Amy S. Sturt, Emily L. Webb, Catriona Patterson, Comfort R. Phiri, Tobias Mweene, Eyrun F. Kjetland, Maina Mudenda, Joyce Mapani, Mable M. Mutengo, James Chipeta, Govert J. van Dam, Paul L. A. M. Corstjens, Helen Ayles, Richard J. Hayes, Isaiah Hansingo, Piet Cools, Lisette van Lieshout, Helena Helmby, Grace A. McComsey, Suzanna C. Francis, Amaya L. Bustinduy

**Affiliations:** ^1^Department of Clinical Research, London School of Hygiene and Tropical Medicine, London, United Kingdom; ^2^MRC International Statistics and Epidemiology Group, London School of Hygiene and Tropical Medicine, London, United Kingdom; ^3^Department of Infection Biology, London School of Hygiene and Tropical Medicine, London, United Kingdom; ^4^Zambart, Lusaka, Zambia; ^5^Department of Infectious Diseases, Oslo University Hospital, Oslo, Norway; ^6^University of KwaZulu-Natal, Discipline of Public Health, Durban, South Africa; ^7^Department of Obstetrics and Gynecology, Livingstone Central Hospital, Livingstone, Zambia; ^8^Institute of Basic and Biomedical Sciences, Levy Mwanawasa Medical University, Lusaka, Zambia; ^9^Department of Pediatrics, University of Zambia, Lusaka, Zambia; ^10^Department of Parasitology, Leiden University Medical Center, Leiden, Netherlands; ^11^Department of Cell and Chemical Biology, Leiden University Medical Center, Leiden, Netherlands; ^12^Faculty of Medicine and Health Sciences, Ghent University, Ghent, Belgium; ^13^University Hospitals Cleveland Medical Center and Case Western Reserve University, Department of Pediatrics and Medicine, Cleveland, OH, United States

**Keywords:** HIV-1, female genital schistosomiasis, S. haematobium, inflammation, sub-Saharan Africa, genital tract, cervicovaginal lavage (CVL), polymerase chain reaction (PCR)

## Abstract

HIV-1 infection disproportionately affects women in sub-Saharan Africa, where areas of high HIV-1 prevalence and *Schistosoma haematobium* endemicity largely overlap. Female genital schistosomiasis (FGS), an inflammatory disease caused by *S. haematobium* egg deposition in the genital tract, has been associated with prevalent HIV-1 infection. Elevated levels of the chemokines MIP-1α (CCL-3), MIP-1β (CCL-4), IP-10 (CXCL-10), and IL-8 (CXCL-8) in cervicovaginal lavage (CVL) have been associated with HIV-1 acquisition. We hypothesize that levels of cervicovaginal cytokines may be raised in FGS and could provide a causal mechanism for the association between FGS and HIV-1. In the cross-sectional BILHIV study, specimens were collected from 603 female participants who were aged 18–31 years, sexually active, not pregnant and participated in the HPTN 071 (PopART) HIV-1 prevention trial in Zambia. Participants self-collected urine, and vaginal and cervical swabs, while CVLs were clinically obtained. Microscopy and *Schistosoma* circulating anodic antigen (CAA) were performed on urine. Genital samples were examined for parasite-specific DNA by PCR. Women with FGS (n=28), defined as a positive *Schistosoma* PCR from any genital sample were frequency age-matched with 159 FGS negative (defined as negative *Schistosoma* PCR, urine CAA, urine microscopy, and colposcopy imaging) women. Participants with probable FGS (n=25) (defined as the presence of either urine CAA or microscopy in combination with one of four clinical findings suggestive of FGS on colposcope-obtained photographs) were also included, for a total sample size of 212. The concentrations of 17 soluble cytokines and chemokines were quantified by a multiplex bead-based immunoassay. There was no difference in the concentrations of cytokines or chemokines between participants with and without FGS. An exploratory analysis of those women with a higher FGS burden, defined by ≥2 genital specimens with detectable *Schistosoma* DNA (n=15) showed, after adjusting for potential confounders, a higher Th2 (IL-4, IL-5, and IL-13) and pro-inflammatory (IL-15) expression pattern in comparison to FGS negative women, with differences unlikely to be due to chance (p=0.037 for IL-4 and p<0.001 for IL-5 after adjusting for multiple testing). FGS may alter the female genital tract immune environment, but larger studies in areas of varying endemicity are needed to evaluate the association with HIV-1 vulnerability.

## Introduction

HIV-1 infection disproportionately affects women in sub-Saharan Africa (1), where areas of high HIV-1 prevalence and *Schistosoma haematobium* endemicity largely overlap (2). Female genital schistosomiasis (FGS), caused most frequently by *S. haematobium* egg deposition in the genital tract, has been associated with prevalent HIV-1 infection in cross-sectional studies (3). The presence of *S. haematobium* eggs in genital tissue is also associated with vascularization (4) and the accumulation of CD4+ lymphocytes and macrophages (5), making the granuloma-associated environment a potential contributor to HIV-1 vulnerability. In addition to modulation of the local cervicovaginal environment, FGS has also been associated with a higher frequency of systemic CD4 T-cells expressing the chemokine receptor CCR5 (6). Tissue-entrapped eggs are also associated with clinically visible FGS-associated manifestations in the cervicovaginal mucosa (7). FGS lesions may breach the intact cervicovaginal immune barrier and are hypothesized to provide an entry point for HIV-1 infection (2, 3). However, the underlying mechanism for potential HIV-1 vulnerability in FGS has not been fully characterized and requires further investigation.

The presence of *S. haematobium* eggs in human tissue commonly provokes an inflammatory response (5). Cervicovaginal inflammation has emerged as an important risk factor for HIV-1 acquisition, with the presence of increased chemotactic cytokine concentrations, specifically macrophage inflammatory protein-1α (MIP-1α [CCL-3]), MIP-1ß [CCL-4], interleukin (IL)-8 [CXCL-8], and interferon-γ inducible protein-10 (IP-10 [CXCL-10]), conferring increased risk (8). Broadly, while pro-inflammatory cytokines are central in recruiting and activating HIV-1 target cells, they also propagate a cascade of downstream cellular processes that enact functions central to HIV-1 pathogenesis (9). The presence of pro-inflammatory cytokines in the female genital tract may also be associated with HIV-1 replication (via stimulation of transcription factors) (10), an increased frequency and activation of HIV-1 target cells (9, 11, 12) and proteomic signatures suggestive of tissue remodeling that may compromise cervicovaginal barrier function (12).

Sexually transmitted infection (STI) and a “non-optimal” cervicovaginal microbiota contribute to a vaginal pro-inflammatory environment (11, 13), and are thus important risk factors for HIV-1 acquisition (14, 15), but elevated cervicovaginal cytokine and chemokine levels have also been detected in their absence (8). In addition to STI and cervicovaginal microbiota, a number of additional biological and behavioral factors influence the levels of detectable soluble immune proteins in the female genital tract, including the presence of semen (16), cervical ectopy (16), use of hormonal contraception (17), menstrual cycle (18), and intravaginal cleansing practices (13, 17).

FGS may be an unmeasured co-factor contributing to cervicovaginal inflammatory signatures in endemic sub-Saharan African populations (8, 11). *S. haematobium* infection, in the absence of evaluation for genital involvement, has been associated with altered levels of systemic (19) and cervicovaginal cytokines (20). While male genital schistosomiasis has been associated with elevated seminal fluid cytokine concentrations (21), little is known regarding the human cervicovaginal environment in FGS or the role of the immune response. We hypothesize that FGS modulates the cervicovaginal immune environment and that evidence of FGS-associated cervicovaginal inflammation may provide insight into a causal mechanism for the association between FGS and HIV-1. In this cross-sectional study, we evaluate cervicovaginal cytokines and chemokines in women with and without FGS.

## Methods

### Study Setting and Participants

The cross-sectional bilharzia and HIV (BILHIV) study was nested in HPTN 071 (PopART), a cluster randomized trial to measure the impact of an HIV-1 combination prevention package (22). HIV-1 incidence was measured in an HPTN 071 (Pop-ART) Population Cohort comprised of one randomly selected adult (18 to 44 years of age) from a random sample of households in each community who provided data and blood samples at baseline, 12, 24, and 36 months (22). After the 36-month HPTN 071 (PopART) visit, trained community workers made home visits between January and August 2018 to women who had expressed interest in the BILHIV study (23). Women were eligible if they were 18–31 years old, not pregnant, sexually active, and resident in one of the two urban communities that participated in HPTN 071 (PopART) in Livingstone, Zambia. Following written informed consent, the BILHIV study home visit included a questionnaire, genital self-sampling (cervical and vaginal), and a urine specimen, as previously described (23).

### Clinic-Based Sample Collection

Within days of self-sampling, enrolled women who were not currently menstruating were invited to attend Livingstone Central Hospital cervical cancer screening clinic, where one of two trained midwives performed a cervicovaginal lavage (CVL). Cervicovaginal images were captured with a portable colposcope (MobileODT, Tel Aviv, Israel) and were evaluated by one author (EFK) for the presence of any of the four recognized FGS cervicovaginal manifestations: homogenous yellow sandy patches, grainy sandy patches, rubbery papules, and abnormal blood vessels (24). Women having these manifestations (24) and women with any positive urine or genital *Schistosoma* diagnostic were treated free of charge with 40 mg/kg praziquantel. Testing for STI was not performed at the point-of-care and participants with suspected STI were offered syndromic management, as per local guidelines (25).

### CVL Specimen Processing

After speculum insertion, normal saline (10 ml) was flushed continuously with a bulb syringe across the cervix and vaginal walls for 1 min and collected from the posterior fornices. CVL fluid was transferred to a 15 ml conical polypropylene tube and stored temporarily in a refrigerator (4°C) on ice until transfer to the laboratory. Protease inhibitor (Cocktail Set I, Calbiochem, Merck Millipore, Darmstadt, Germany) was added to one 1.5 ml aliquot for cytokine and chemokine testing and stored at −80°C, as previously described (17). Specimens were stored for a maximum of 20 months (range 12–20) and were not previously thawed. After thawing, specimens were centrifuged at 320g for 10 min and the supernatant removed. CVL color was visually assessed and a 10 μl aliquot was placed on a Hemastix test strip (Siemens, Erlangen, Germany). As per the manufacturer’s instructions, CVL hemoglobin concentrations were recorded after comparing the test strip with color categories representing approximate quantities of erythrocytes (ery) per µL: none, trace, low (25 ery/μL), moderate (80 ery/μL), high (200 ery/μL) (17).

### Multiplex Bead Based Assays

Luminex MAGPIX^©^ was used to measure concentrations of seventeen soluble cytokines and chemokines using MILLIPLEX Human Cytokine/Chemokine Magnetic Bead kits (Merck Millipore, Darmstadt, Germany) according to the manufacturer’s instructions and recommendations for dilute samples, i.e. CVL. The concentrations of eotaxin (CCL-11), interferon-gamma (IFN-γ), IL-10, IL-13, IL-15, IL-17A, IL-1α, IL-1β, IL-4, IL-5, IL-6, IL-8 (CXCL-8), IP-10 (CXCL-10), monocyte chemoattractant protein (MCP-1) (CCL-2), MIP-1α, (CCL-3), MIP-1β (CCL-4) and tumor necrosis factor-α (TNF-α) were measured in undiluted CVL in duplicate. The lower limit of detection was between 0.26 and 5.66 pg/ml for the 17 cytokines and chemokines measured (**S1 Table**). Using a Luminex MAGPIX^©^ bioanalyzer and xPONENT software (version 4.2), the median fluorescent intensity was measured, background-adjusted, and converted into analyte concentrations using a 5 parameter logistic regression equation to interpolate standard curves. To minimize between-plate variations in cytokine and chemokine concentrations, two specimen controls were included in duplicate across plates and equal proportions of specimens with FGS, *probable* FGS, and FGS *negative* were distributed across six 96-well plates (8). Cytokine or chemokine concentrations below the lower limit of quantification (LLOQ) were imputed to be the midpoint of the lowest concentration for each analyte and zero and concentrations above the upper limit of quantification were imputed as the highest concentration for each analyte.

### HIV-1

Laboratory-based fourth-generation HIV-1 testing (Abbott Architect HIV Ag/Ab Combo Assay). was performed for HPTN 071 (PopART) Population Cohort participants at each study visit (22).

### Circulating Anodic Antigen

A lateral flow assay utilizing up-converting reporter particles for the quantification of CAA was performed on urine samples at the Leiden University Medical Center (LUMC), as previously described (23, 26). CAA levels reflect the burden of live schistosomes and decline after successful treatment with praziquantel (27, 28). Analyzing the equivalent of 417 µl urine (wet reagent, UCAA***hT***417), a CAA value of >0.6 pg/ml was considered positive (28).

### PCR for Detection of *Schistosoma* DNA

DNA extraction and PCR set up was performed at LUMC, using a custom automated liquid handling station (Hamilton, Switzerland), as previously described (23). DNA was extracted from 200 µl of specimen (cervical swab, vaginal swab, CVL): with QIAamp spin columns (QIAGEN Benelux; Venlo, The Netherlands). Detection of the schistosome-specific internal-transcribed-spacer-2 (ITS2) target was performed by real-time PCR as previously described (23, 29). This PCR does not differentiate between *Schistosoma* species. DNA amplification and detection were performed with the CFX96 Real Time PCR Detection System (BioRad, California, USA). The output in cycle quantification value (Cq), reflecting the parasite-specific DNA load in the tested sample, was analyzed using BioRad CFX software. Parasite DNA loads were categorized by the following pre-specified Cq thresholds: high (Cq<30), moderate (30≤ Cq <35), low (35≤ Cq <50) and negative (no Cq detected), as previously described (30).

### STI Detection

We quantified *Chlamydia trachomatis*, *Neisseria gonorrhoeae*, *Mycoplasma genitalium*, and *Trichomonas vaginalis* using the S-DiaCTNG™ (for *C. trachomatis* and *N. gonorrhea*) and S-DiaMGTV™ (for *M. genitalium* and *T. vaginalis*) (both Diagenode Diagnostics, Seraing, Belgium) on DNA obtained from cervical swabs at Ghent University (Ghent, Belgium) according to the manufacturer’s instructions. Amplification was carried out on the LightCycler480^®^ and the LightCyclerR 480 Software Version 1.5 (Roche, Basel, Switzerland). To quantify each of the target species, standard curves were constructed from a tenfold dilution series of DNA from *C. trachomatis*, *N. gonorrhoeae*, *M. genitalium*, and T. vaginalis. Genomic DNA of *C. trachomatis* ATCC VR-571B, *T. vaginalis* ATCC 50148 and *M. genitalium* G37 was obtained from the American Type Culture Collection (ATCC). Genomic DNA from *N. gonorrhoeae* was obtained after culturing strain ATCC 43069 at 35°C ±1°C for 5 days on chocolate agar (Becton Dickinson) and extracting DNA from colonies using Roche High Pure DNA Purification kit (Roche). All DNA concentrations were determined using NanoDrop (Thermo Fisher scientific, Erembodegem, Belgium). The genomic concentrations were calculated using the described genomic sizes of the type strains. Both the standard curves and samples were run in duplicate. The number of bacteria and protozoan concentration was expressed as genome equivalents per ml (geq/ml) (31).

### Ethical Considerations

The study was approved by the University of Zambia Biomedical Research Ethics Committee (reference 011-08-17), the Zambia National Health Research Authority and the London School of Hygiene and Tropical Medicine Ethics Committee (reference 14506). Permission to conduct the study was given by Livingstone District Health Office and the superintendent of Livingstone Central Hospital.

### FGS Definitions

**Figure 1:**
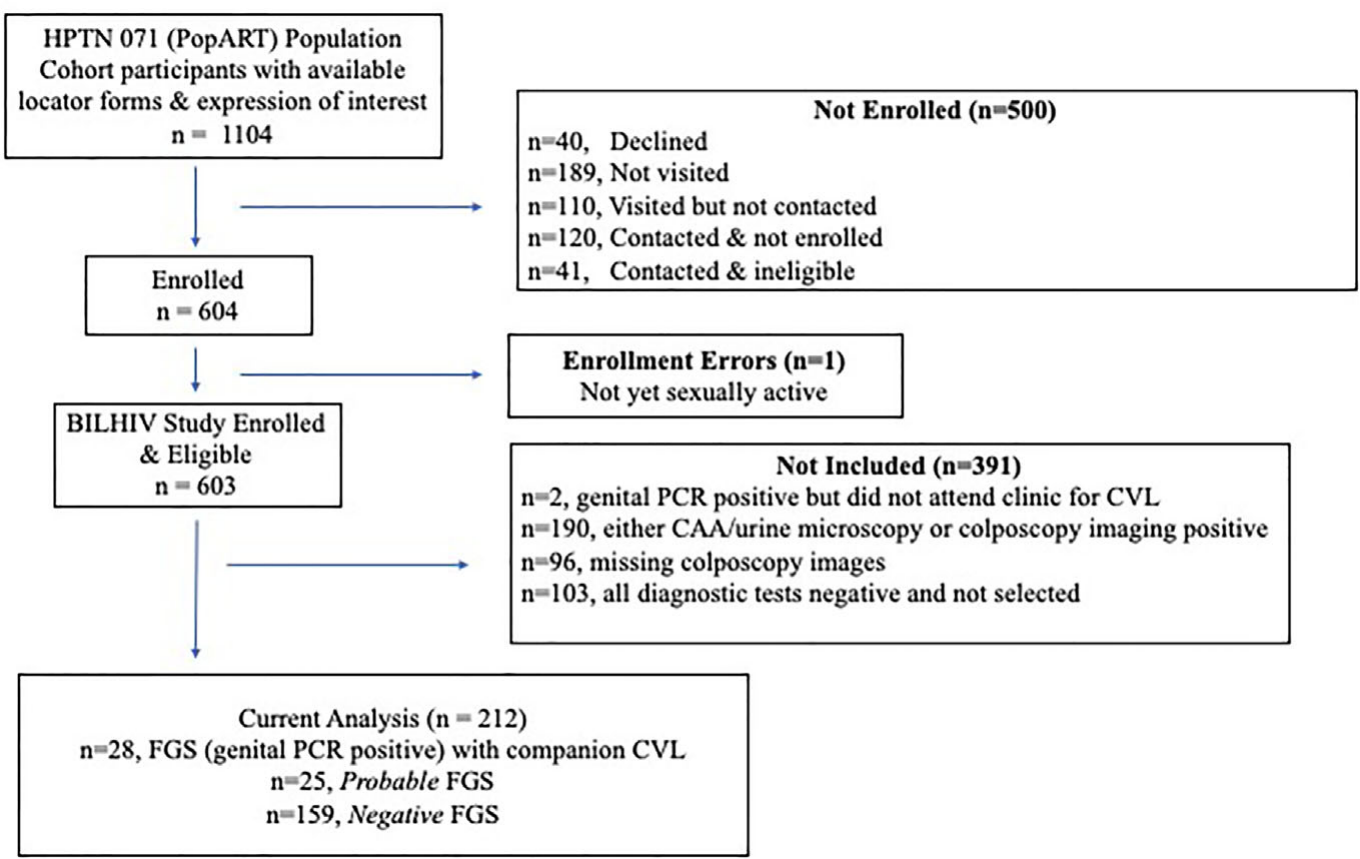
Study Flow Diagram. Not visited (n=189)– the participant was not visited before the study closed for enrollment; Visited but not contacted (n=110)– a visit was made to the study household, but the participant could not be located (70), had relocated (39), or died (1); Contacted & not immediately enrolled (n=120)– visited but not recruited (42), out of town (18), declined to participate (60); Contacted & ineligible (n=41)– virgin (16), pregnant (17), over 31 (8).

The FGS categories were defined by the results of four investigations: *Schistosoma* PCR (on DNA extracted from cervical swabs, vaginal swabs, or CVL), colposcopy image review, urine CAA, and urine microscopy. Participants were grouped by the outcomes of their diagnostic tests into three mutually exclusive categories. FGS was defined as at least one positive *Schistosoma* PCR on a genital specimen (cervical swab, vaginal swab and/or CVL). In participants with a negative *Schistosoma* PCR, *probable* FGS was defined as the presence of urinary schistosomiasis, detected with either urine CAA or urine microscopy, in combination with one of four clinical findings suggestive of FGS on any colposcope-obtained photograph (24). FGS *negative* was defined as negative results on all diagnostic assays. Participants with results for all available diagnostic tests who were *Schistosoma* genital PCR negative and did not qualify for the FGS, *probable* FGS, or FGS *negative* groups (n=190) were not eligible for study inclusion (**Figure 1**). All participants with FGS (n=28) who attended clinic follow up and provided a CVL specimen and all participants with *probable* FGS (n=25) were selected for measurement of cytokines and chemokines in CVL samples. Three FGS *negative* participants were selected for every FGS and *probable* FGS participant, using a random number generator. The FGS *negative* participants were frequency matched by age to the participants with FGS.

## Statistical Methods

Participant characteristics were summarized by median and interquartile range (IQR) for continuous variables, and by frequency and percentage for categorical variables. Differences in characteristics between the FGS categories were evaluated using Fisher’s exact and chi-squared tests. For cytokines or chemokines with at least 70% of sample results above the LLOQ, differences in median cytokine or chemokine concentrations between FGS categories were evaluated using the Wilcoxon-Mann-Whitney test. Cytokines or chemokines with <70% of the sample results above the LLOQ were analyzed as binary variables (presence/absence) and compared between FGS categories using Fisher’s exact and chi-squared tests. To correct for multiple comparisons we used a Monte Carlo simulation approach with 1000 replicates (32): for each replicate the labels of the groups being compared were randomly permuted and statistical tests were repeated, to generate empirical p-values. For further analysis, cytokine or chemokine concentrations with at least 70% of sample results above the LLOQ were log-transformed to normalize their distribution, and linear regression was performed to evaluate the association between FGS and mean cytokines or chemokine in univariable and multivariable analysis and expressed with 95% confidence intervals. For the cytokines with <70% of the sample results above the LLOQ (IL-5, IL-13, IL-15, and TNF-α), logistic regression was used to evaluate the association between FGS and detectable cytokine concentrations and expressed with 95% confidence intervals. To adjust for potential confounders, we developed a causal conceptual framework (**S1 Figure**) to inform our minimal adjustment set. For the cytokines and chemokines analyzed by linear regression, we adjusted for age, education, community of residence, and the presence of any STI. Since hormonal contraception is associated with the outcome, but not the exposure (FGS), it was also included in the multivariable model to improve precision. For the cytokines analyzed by logistic regression (IL-5, IL-13, IL-15, and TNF-α), due to the relatively low number of participants with concentrations above the LLOQ, these cytokines were adjusted for age and STI. Because HIV-1 status and the presence of hemoglobin in CVL are both potentially influenced by FGS (2), and may also affect CVL cytokine or chemokine concentrations, these variables were considered to be on the causal pathway and were not included in the final multivariable model (**S1 Figure**).

Our primary hypothesis was that FGS modulates the cervicovaginal microenvironment with a secondary hypothesis that FGS may increase the concentration of selected HIV-1 acquisition associated chemokines (8). Thus, our primary analysis focused on the detection of *Schistosoma* DNA in the genital tract, comparing FGS vs FGS *negative* participants. As a secondary aim, participants with FGS and *probable* FGS were combined and compared with the FGS *negative* group.

Spearman’s rank correlation was used to evaluate the strength of the relationship between individual analytes (**S2 Figure**). Since many cytokines and chemokines were correlated, we performed Principal Components Analysis (PCA) on the log-transformed analyte concentrations to generate new uncorrelated “components” that were linear combinations of the initial variables. The first two principal components captured the majority of the variability in the data and were taken forward for additional comparisons between FGS groups (**S2 Table**).

To evaluate the possible association between intensity of FGS presentation and changes in cytokine or chemokine concentrations, two *ad hoc* exploratory analyses were performed: (1) participants with ≥2 genital samples with detectable *Schistosoma* DNA levels were compared with those in the FGS *negative* group, (2) participants with a moderate/high genital *Schistosoma* DNA concentration (defined by a Cq <35 in at least one of the three examined samples) were compared with those in the FGS *negative* group.

In this study we measured the concentration of cytokines and chemokines in CVL. However, the presence of hemoglobin in CVL may serve as a surrogate marker for the presence of systemic and/or menstrual blood in the cervicovaginal environment. HIV-1 status and the presence of hemoglobin in CVL are potentially influenced by FGS (2), but may also independently affect cytokines and chemokine concentrations. Thus, we performed two sensitivity analyses, one compared participants with FGS with those in the FGS *negative* group after excluding the participants who were HIV-1 positive from both groups. A second sensitivity analysis compared participants with FGS with those in the FGS *negative* group after excluding the participants whose CVL sample displayed the presence of hemoglobin. Data were analyzed using STATA 15.1 (Stata Corporation, College Station, TX). P-values less than 0.05 were classified as demonstrating “evidence” of an association and p-values between 0.05 and 0.10 were classified as demonstrating “some evidence” of an association.

## Results

A total of 603 eligible women were enrolled and 212 (35.2%) were included in this study (**Figure 1**). Overall, 13.2% (28/212) of women had FGS, defined by a positive genital *Schistosoma* PCR from any of the following sites: 8.5% (18/212) cervical swab, 6.6% (14/212) vaginal swab, and 6.6% (14/212) CVL. *Probable* FGS was detected in 25 women, and 61.1% (159/262) of the women who were negative on all diagnostic tests were randomly selected for inclusion in this study.

### Baseline Characteristics

The majority of the participants had received at least secondary education, were using hormonal contraception, and had detectable hemoglobin in their CVL. At the conclusion of HPTN 071 (PopART), HIV-1 prevalence was 17.0% (36/212) among the women included in this study and one-third of the women had at least one STI (**Table 1**). Active schistosome infection, defined as either a positive urine microscopy (11.8%, 25/212) or detectable CAA (20.2%, 43/212), was reported in 21.2% (45/212). A small proportion of women reported current water contact, but more than half reported childhood water contact.

**Table 1:** Baseline characteristics of the 212 study participants by female genital schistosomiasis (FGS) status.

Socio-behavioral Characteristics		FGS^*^ % (n = 28)	FGS Probable^*^ % (n = 25)	FGS Negative^*^ % (n = 159)	p-value
Age in years	Median (IQR)	22 (20–24)	27 (23–31)	23 (22–24)	0.001
Marital Status	Single	42.9 (12)	16.0 (4)	45.9 (73)	0.04^∫^
	Married or Cohabitating	57.1 (16)	80.0 (20)	50.9 (81)	
	Divorced or Separated	0.0 (0)	4.0 (1)	3.1 (5)	
Education (highest level)	None/Any Primary School	32.1 (9)	48.0 (12)	22.0 (35)	0.04^∫^
	Any Secondary School	67.9 (19)	52.0 (13)	70.4 (112)	
	Training in a Trade	0.0 (0)	0.0 (0)	7.6 (12)	
Employment Status	Working	14.3 (4)	44.0 (11)	25.8 (41)	0.05
	Not Working	85.7 (24)	56.0 (14)	74.2 (118)	
Current Water Contact	None	100.0 (28)	84.0 (21)	86.8 (138)	0.1^∫^
	Any	0.0 (0)	16.0 (4)	13.2 (21)	
Childhood Water Contact	None	14.3 (4)	24.0 (6)	32.1 (51)	0.1
	Any	85.7 (24)	76.0 (19)	67.9 (108)	
Community of Residence	Community A	75.0 (21)	80.0 (20)	41.5 (66)	<0.001
	Community B	25.0 (7)	20.0 (5)	58.5 (93)	
**Sexual behavior characteristics and STI**					
Ever pregnant	No^#^	7.1 (2)	4.0 (1)	17.0 (27)	0.3^∫^
	Yes	92.9 (26)	96.0 (24)	82.4 (131)	
Age at sexual debut	Median (IQR)	16 (15–18)	17 (15–18)	17 (16–18)	0.09
Lifetime sexual partners	Median (IQR)	3 (2–4.5)	2 (1–3)	2 (1–4)	0.3
Currently Sexually Active	No^##^	10.7 (3)	8.0 (2)	16.5 (26)	0.6^∫^
	Yes	89.3 (25)	92.0 (23)	83.5 (132)	
Contraceptive Method	Implant	7.1 (2)	8.0 (2)	8.8 (14)	1.0^∫^
	Injectable	53.6 (15)	48.0 (12)	47.8 (76)	0.9
	Oral Contraceptive Pill	3.6 (1)	12.0 (3)	6.3 (10)	0.5^∫^
	Condoms	10.7 (3)	20.0 (5)	16.4 (26)	0.7^∫^
HIV-1	Not Detected	82.1 (23)	80.0 (20)	83.7 (133)	0.9
	Detected	17.9 (5)	20.0 (5)	16.4 (26)	
*Chlamydia trachomatis*	Not Detected	89.3 (25)	96.0 (24)	91.8 (146)	0.7^∫^
	Detected	10.7 (3)	4.0 (1)	8.2 (13)	
*Neisseria gonorrhea*	Not Detected	100.0 (28)	96.0 (24)	92.4 (147)	0.4^∫^
	Detected	0.0 (0)	4.0 (1)	7.6 (12)	
*Mycoplasma genitalium*	Not Detected	100.0 (28)	96.0 (24)	95.6 (152)	0.5^∫^
	Detected	0.0 (0)	4.0 (1)	4.4 (7)	
*Trichomonas vaginalis*	Not Detected	67.9 (19)	68.0 (17)	78.6 (125)	0.3
	Detected	32.1 (9)	32.0 (8)	21.4 (34)	
Any STI	Not Detected	64.3 (18)	56.0 (14)	67.3 (107)	0.5
	Detected	35.7 (10)	44.0 (11)	32.7 (52)	
**Clinical Findings**					
Hemoglobin in CVL (ery/µL)^ϑ^	None	28.6 (8)	25.0 (6)	36.1 (57)	0.3^∫^
	Trace	17.9 (5)	12.5 (3)	15.2 (24)	
	25	32.1 (9)	16.7 (4)	17.7 (28)	
	80	3.6 (1)	16.7 (4)	17.1 (27)	
	200	17.9 (5)	29.2 (7)	13.9 (22)	
Colposcopy Findings^ϑϑ^	Sandy Patches	22.2 (6)	76.0 (19)	0.0 (0)	N/A
	Rubbery Papule	0.0 (0)	0.0 (0)	0.0 (0)	
	Abnormal Blood Vessels	22.2 (6)	24.0 (6)	0.0 (0)	
	No FGS findings	56.0 (15)	0.0 (0)	100.0 (159)	
					

^*^FGS – Schistosoma PCR positive specimen from cervicovaginal lavage, vaginal swab or cervical swab; probable FGS– Schistosoma PCR negative and either positive circulating anodic antigen (CAA) or urine microscopy and suggestive expert-reviewed colposcopy imaging; FGS negative – negative Schistosoma PCR and negative CAA and negative urine microscopy and negative expert-reviewed colposcopy imaging.

^∫^Fisher’s exact test.

^#^Self-reported history of ever having a pregnancy, participants reporting “no answer” (n=1, FGS negative group) are not shown.

^##^Participants reporting “no answer” (n=1, FGS negative group) are not shown.

^ϑ^Hemoglobin was measured with Hemastix® test strips using a color chart measured in erythrocytes (ery) per µl.

^ϑϑ^Colposcopy findings were included in the study inclusion criteria, so no p-value is shown; one participant with FGS did not have interpretable colposcopy results. Images were interpreted based on the presence of sandy patches or rubbery papules, if these findings were present, the additional finding of abnormal blood vessels was not noted.

There was strong evidence of a difference in community of residence between FGS, *probable* FGS, and FGS *negative* participants (p=0.001) with participants with FGS and *probable* FGS more likely to live in Community A than participants in the FGS *negative* group (**Table 1**). There were differences between the three categories of FGS status for age (p<0.001), educational attainment (p=0.04), employment (p=0.05), and marital status (p=0.04) with participants in the *probable* FGS group more likely to be older, have a primary school education, be employed, and be married than FGS and FGS *negative* participants. Other characteristics were similar by FGS status.

### Expression Profiles of Cytokines and Chemokines in CVL

**Figure 2:**
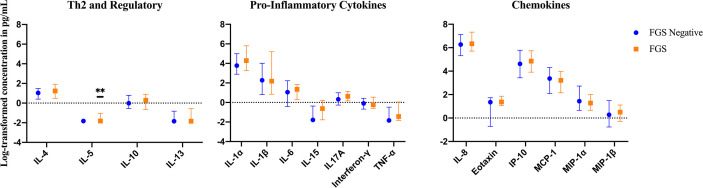
Median with interquartile range of the log-transformed crude concentrations of eleven cytokines and six chemokines in cervicovaginal lavage of participants with (FGS: n=28) and without female genital schistosomiasis (FGS negative: n=159) ^χ^. FGS – *Schistosoma* PCR positive specimen from cervicovaginal lavage, vaginal swab or cervical swab; FGS *negative* – negative genital PCR and negative circulating anodic antigen and negative urine microscopy and negative expert-reviewed colposcopy imaging. ^χ^ p-value after adjustment for multiple testing with a Monte-Carlo simulation approach, p=0.14 p-value symbol legend: **p < 0.05.

The mean, median and range of concentrations (pg/ml) of the 17 cytokines and chemokines measured are displayed in **S1 Table**. The distributions of log-transformed concentrations (median and interquartile range) of the 17 cytokines and chemokines are displayed by FGS status in **Figure 2**.

### FGS Signature - Crude and Adjusted Expression Profiles

**Figure 3:**
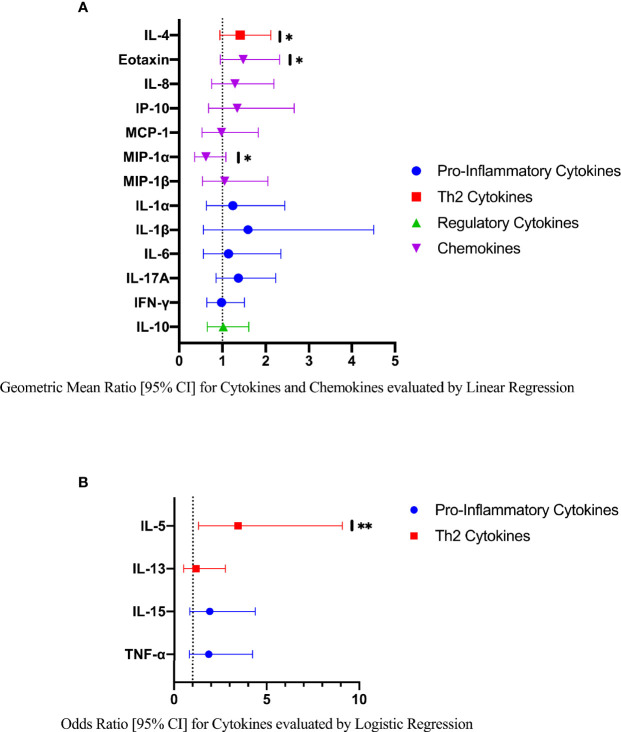
Comparison of the concentration or presence of eleven cytokines and six chemokines in cervicovaginal lavage of participants with (FGS, n=28) and without (FGS negative, n=159) female genital schistosomiasis^χ^. **(A)** Concentrations of eotaxin, IFN-γ, IL-1α, IL-1β, IL-4, IL-6, IL-8, IL-10, MCP-1, MIP-1α, and MIP-1β were compared between FGS and FGS negative participants by linear regression and adjusted for age, community of residence, education, presence of sexually transmitted infection and hormonal contraceptive use, with results shown as geometric mean ratios with 95% CI. **(B)** Presence/absence of IL-5, IL-13, IL-15 and TNF-α were compared by logistic regression and adjusted for age and sexually transmitted infection, with results shown as odds ratio with 95% CI. The line at 1 indicates the value at which there is no difference between the FGS and FGS *negative* groups. p-value symbol legend: *p < 0.1 **p < 0.05. **^χ^**female genital schistosomiasis – *Schistosoma* PCR positive specimen from cervicovaginal lavage, vaginal swab or cervical swab; FGS *negative* – negative genital PCR and negative urine circulating anodic antigen and negative urine microscopy and negative expert-reviewed colposcopy imaging.

Compared to FGS *negative* women, IL-5 was elevated in participants with FGS (**Table 2**, **Figure 2**, crude p-value 0.02, p-value after adjustment for multiple testing 0.14). Compared to FGS *negative* women, women with FGS had similar expression profiles of chemokines that predicted HIV-1 acquisition risk in a South African study (MIP-1α [CCL-3], MIP-1β [CCL-4], IL-8 [CXCL-8], and IP-10 [CXCL-10]) (8). This was confirmed after adjusting for age, STI, community of residence, education, and use of hormonal contraception (**Figure 3**). Principal Components Analysis identified that two Principal Components accounted for 60.0% of the variability in the data (**S2 Table**). Taken forward, there was no difference in mean scores for these two Principal Components by FGS status (**S3 Figure**).

**Table 2:** Crude and adjusted associations (with 95% confidence intervals) of FGS^*^ status with concentrations of cytokines and chemokines in cervicovaginal lavage^**^.

Analyte	%(n) above LLOQ	FGS Negative ^‡^n=159	FGS n=28	p-value^‡‡^	GMR FGS vs FGS Negative (n=28) ^†^	p-value ^† †^
**Linear Regression** *- Analytes with >70% above LLOQ*						
Eotaxin	73.6 (156)	3.85	3.92	0.22	1.48 (0.95–2.32)	0.08
Interferon–γ	84.0 (178)	0.91	0.80	0.71	0.98 (0.64–1.51)	0.94
IL–10	90.1 (191)	0.98	1.33	0.75	1.02 (0.65–1.61)	0.93
IL–17A	93.4 (198)	1.38	1.86	0.14	1.37 (0.85–2.23)	0.64
IL1–α	99.5 (211)	44.00	73.08	0.17	1.24 (0.63–2.44)	0.19
IL1–β	93.4 (198)	9.79	9.05	0.38	1.59 (0.56–4.51)	0.52
IL–4	96.2 (204)	2.79	3.42	0.20	1.41 (0.94–2.12)	0.09
IL–6	87.7 (186)	2.85	3.81	0.67	1.14 (0.56–2.35)	0.71
IL–8	100.0 (212)	526.03	566.68	0.33	1.29 (0.75–2.19)	0.34
IP–10	100.0 (212)	101.86	128.38	0.42	1.34 (0.68–2.66)	0.39
MCP–1	100.0 (212)	29.04	24.68	0.79	0.98 (0.53–1.83)	0.95
MIP1–α	98.1 (208)	4.23	3.61	0.15	0.62 (0.36–1.08)	0.09
MIP1–β	84.0 (177)	1.32	1.64	0.67	1.05 (0.54–2.05)	0.89
**Logistic Regression -** *Analytes with <70% above LLOQ*						
**Analyte**	**%(n) above LLOQ**	**FGS Negative** **%(n=159)**	**FGS** **%(n=28)**	**p-value^χ^**	**OR FGS vs FGS Negative^χχ^**	**p-value**^#^
IL–5	17.0 (36)	13.84 (22)	32.1 (9)	0.02	3.44 (1.30–9.05)	0.02
IL–13	32.6 (69)	32.7 (52)	35.7 (10)	0.76	1.17 (0.50–2.75)	0.72
IL–15	47.2 (100)	44.7 (71)	60.7 (17)	0.12	1.91 (0.84–4.36)	0.12
TNF–α	41.0 (87)	35.9 (57)	50.0 (14)	0.15	1.85 (0.81–4.22)	0.14

FGS, female genital schistosomiasis; GMR, geometric mean ratio; LLOQ, lower limit of quantification.

^*^FGS – Schistosoma PCR positive specimen from cervicovaginal lavage, vaginal swab or cervical swab; FGS negative – negative Schistosoma PCR and negative circulating anodic antigen and negative urine microscopy and negative expert-reviewed colposcopy imaging.

^**^ Concentrations are reported in pg/ml.

^‡^n=25 Participants with probable FGS are not shown.

^‡‡^ Rank sum p-value.

^†^Adjusted for age, STI, educational level attained, community of residence, and hormonal contraception.

^††^F-test p-value.

^χ^ Chi-squared p-value.

^χχ^ Adjusted for age and STI.

^#^Likelihood ratio test p-value.

When the FGS and *probable* FGS groups were combined, in the crude analysis (**S4 Figure**) and after adjustment for possible confounders, participants with FGS/*probable* FGS had higher concentrations of TNF-α than FGS *negative* participants (**S5 Figure**, p=0.03, p-value adjusted for multiple testing 0.09).

### Exploratory Analyses – Clinical Disease Burden

**Figure 4:**
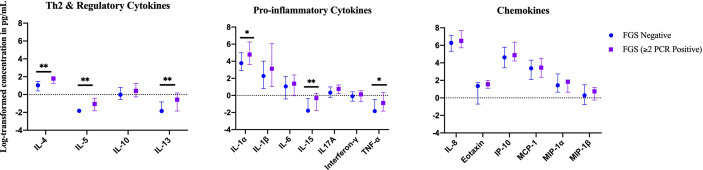
Median with interquartile range of the log-transformed crude concentrations of eleven cytokines and six chemokines in cervicovaginal lavage by FGS^χ^ burden (*Schistosoma* PCR in ≥ two genital specimens: n=15, FGS negative: n=159) ^χχ^. ^χ^FGS – *Schistosoma* PCR positive specimen from cervicovaginal lavage, vaginal swab or cervical swab; FGS *negative* – negative *Schistosoma* PCR and negative circulating anodic antigen and negative urine microscopy and negative expert-reviewed colposcopy imaging. ^χχ^p-value after adjustment for multiple testing with a Monte-Carlo simulation approach, p < 0.001. p-value legend *p < 0.1 **p < 0.05.

**Figure 5:**
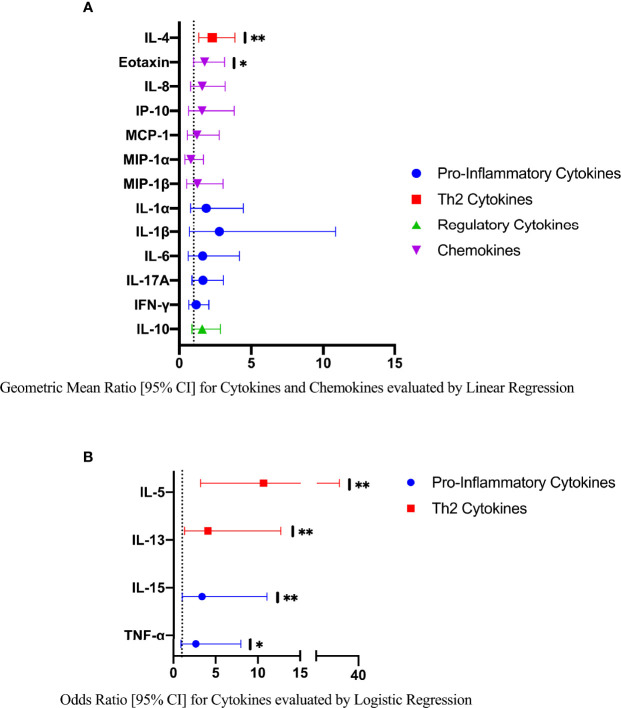
Comparison of the concentrations or presence of eleven cytokines and six chemokines in cervicovaginal lavage in participants with greater FGS burden (*Schistosoma* PCR positive in ≥ two genital specimens, n=15) and participants without female genital schistosomiasis (n=159) ^χ^. **(A)** Concentrations of eotaxin, IFN-γ, IL-1α, IL-1β, IL-4, IL-6, IL-8, IL-10, MCP-1, MIP-1α, and MIP-1β were compared between FGS and FGS *negative* participants by linear regression adjusted for age, community of residence, education, presence of sexually transmitted infection and hormonal contraceptive use, with results shown as geometric mean ratios with 95% CI. **(B)** Presence/absence of IL-5, IL-13, IL-15 and TNF-α were compared by logistic regression and adjusted for age and sexually transmitted infection, with results shown as odds ratio with 95% CI. The line at 1 indicates the value at which there is no difference between the FGS and FGS *negative* groups. p-value symbol legend: *p <0.1 **p <0.05. **^χ^**female genital schistosomiasis – *Schistosoma* PCR positive specimen from cervicovaginal lavage, vaginal swab or cervical swab; FGS *negative* – negative *Schistosoma* PCR and negative circulating anodic antigen and negative urine microscopy and negative expert-reviewed colposcopy imaging.

In an exploratory analysis of participants (n=15) with a higher FGS burden defined as ≥2 *Schistosoma* PCR positive genital specimens, there was evidence of an elevated concentration of cytokines IL-4, IL-5, IL-13, IL-15 compared to FGS *negative* women (**Figure 4**). This association remained after adjusting for potential confounders (**Figure 5**). After adjustment for multiple comparisons strong evidence remained that IL-4 (multiple testing adjusted p=0.037) and IL-5 (multiple testing adjusted p<0.001) were associated with FGS.

### Exploratory Analyses – *Schistosoma* DNA Concentration

**Figure 6:**
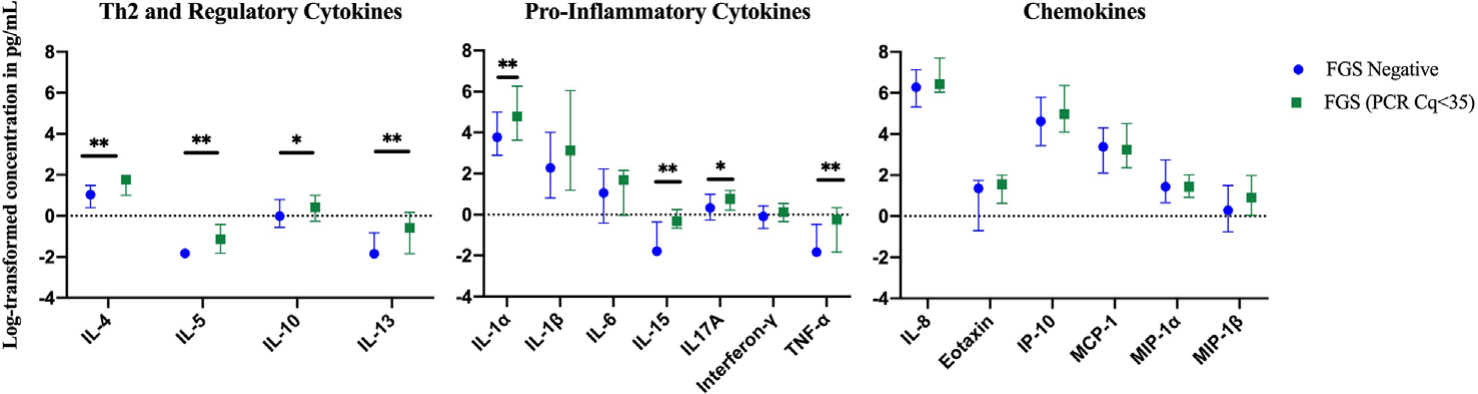
Median with interquartile range of the log-transformed crude concentrations of eleven cytokines and six chemokines in cervicovaginal lavage by FGS burden (*Schistosoma* DNA concentration Cq <35 in any genital specimen, n=15, FGS *negative*:n=159) ^χ^. FGS – *Schistosoma* PCR positive specimen from cervicovaginal lavage, vaginal swab or cervical swab; FGS *negative* – negative *Schistosoma* PCR and negative circulating anodic antigen and negative urine microscopy and negative expert-reviewed colposcopy imaging. ^χ^p-value after adjustment for multiple testing with a Monte-Carlo simulation approach, p=0.001 p-value symbol legend: *p < 0.1 **p < 0.05.

**Figure 7:**
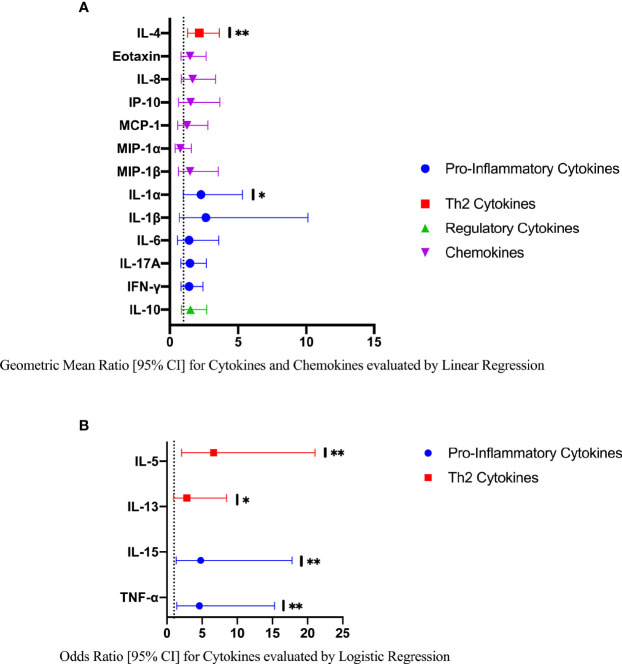
Comparison of the concentration or presence of eleven cytokines and six chemokines in cervicovaginal lavage in participants with moderate to high *Schistosoma* DNA concentrations (Cq <35 in any genital specimen, n=15) and participants without female genital schistosomiasis (n=159) ^χ^. **(A)** Concentrations of eotaxin, IFN-γ, IL-1α, IL-1β, IL-4, IL-6, IL-8, IL-10, MCP-1, MIP-1α, and MIP-1β were compared by linear regression adjusted for age, community of residence, education, presence of sexually transmitted infection and hormonal contraceptive use. Results are represented by geometric mean ratios with 95% CI. **(B)** Presence/absence of IL-5, IL-13, IL-15 and TNF-α were compared by logistic regression and adjusted for age and sexually transmitted infection. Results are indicated by odds ratio with 95% CI. The line at 1 indicates the value at which there is no difference between the FGS and FGS *negative* groups. p-value symbol legend: * <0.1, ** <0.05. ^χ^Female genital schistosomiasis – *Schistosoma* PCR positive specimen from cervicovaginal lavage, vaginal swab or cervical swab; FGS *negative* – negative *Schistosoma* PCR and negative circulating anodic antigen and negative urine microscopy and negative expert-reviewed colposcopy imaging.

In a further exploratory analysis of women (n=15) with FGS and moderate or high genital *Schistosoma* DNA concentration (*Schistosoma* PCR Cq<35) there was evidence that the concentrations of cytokines IL-1α, IL-4, IL-5, IL-13, IL-15, and TNF-α were elevated in participants with *Schistosoma* PCR Cq<35 compared to FGS *negative* women (**Figure 6**). After adjustment for potential confounders, evidence remained that IL-4, IL-5, IL-15, and TNF-α were elevated in participants with moderate/high *Schistosoma* DNA concentration compared to FGS *negative* women (**Figure 7**). After adjustment for multiple comparisons, strong evidence remained that the associations for IL-5 (multiple testing adjusted p=0.001) and TNF-α (multiple testing adjusted p=0.045) were unlikely to have occurred by chance. When comparing the participants with moderate/high *Schistosoma* DNA concentration and the participants with ≥2 PCR positive genital specimens, 11 women overlapped between groups.

### Sensitivity Analyses – HIV-1 and CVL Hemoglobin

We performed sensitivity analyses removing participants with HIV-1 infection or the presence of hemoglobin in CVL from the comparison between FGS and *negative* FGS groups. When n=31 participants with HIV-1 from the FGS and FGS *negative* groups were excluded from the analysis, there was no difference in the expression profiles of cytokines or chemokines in women with FGS compared to FGS *negative* women (**S6 Figure**). When n=139 participants with any detectable hemoglobin in CVL were removed from the analysis (**S7 Figure**), there was some evidence that concentrations of IL-17A and IL-8 (CXCL-8) were higher in participants with FGS (n=8) compared to the FGS *negative* group (n=57) (p=0.13 after allowing for multiple testing).

## Discussion

This study is the first to describe expression patterns of cytokines and chemokines in human FGS, diagnosed by *Schistosoma* PCR from vaginal swabs, cervical swabs, and CVL. The immune environment in helminth infection is often characterized as T helper 2 (Th2) biased and involves the orchestration of cytokines (IL-4, IL-5, IL-13), antibodies, and regulatory cells (33). We did not detect an association between FGS, defined as *Schistosoma* DNA detected in genital PCR specimens, and a change in expression pattern of cytokines or chemokines in CVL including those associated with HIV-1 acquisition in a South African study (8). Compared to the FGS *negative* participants, the Th2 cytokine IL-5 was elevated in the participants with FGS, however after adjustment for multiple comparisons we cannot exclude that this finding may be due to chance.

Previous work on male genital schistosomiasis has shown that infection intensity, defined by seminal egg count, is strongly associated with elevated seminal cytokine concentrations including Th2 (IL-4), regulatory (IL-10), Th1 (IFN-γ) and pro-inflammatory (TNF-α) cytokines (21). Thus, we evaluated FGS burden by performing two exploratory analyses, the results of which show a Th2 expression pattern. First, we investigated the association between multiple PCR-positive genital specimens as a potential proxy marker of higher FGS burden in 15 women with ≥ two positive genital specimens for *Schistosoma* DNA. We also investigated whether *Schistosoma* DNA concentrations in genital samples might be associated with a change in cytokine concentrations in 15 participants with FGS and moderate/high genital *Schistosoma* DNA concentrations (defined as *Schistosoma* PCR Cq<35). After adjusting for potential confounders, the exploratory analyses found a higher cervicovaginal Th2 cytokine response (IL-4, IL5-, IL-13) in participants with ≥ two positive specimens and participants with higher genital *Schistosoma* DNA concentrations. This is not unexpected considering that a Th2 biased immune response is associated with helminth infection (33) and *S. haematobium* exposure in both human and murine hosts (34, 35). There was evidence for higher concentrations of IL-5 after adjustment for multiple comparisons across both exploratory analyses. IL-5 induces eosinophil maturation and an IL-5 response to *Schistosoma* antigens has been associated with microhematuria in children with *S. haematobium* infection (36). There was evidence for higher concentrations of IL-4 after adjustment for multiple comparisons in the exploratory analyses of multiple PCR-positive genital specimens for *Schistosoma* DNA. In the Th2 response, IL-4 directly regulates T-cell differentiation and proliferation (37). The finding of elevated levels of pro-inflammatory TNF-α in the exploratory analyses of participants with moderate/high *Schistosoma* DNA concentrations after adjustment for potential confounders and multiple comparisons, suggests that FGS may promote a mixed Th2 and pro-inflammatory response. TNF-α may be associated with *Schistosoma* granuloma formation (38) and in children with *S. haematobium* infection, TNF-α production has been associated with ultrasound-determined urinary bladder morbidity (39).

We found IL-13 and IL-15 to be associated with a higher clinical FGS burden and higher genital *Schistosoma* DNA concentrations after adjusting for potential confounders, although these associations no longer remained after adjustment for multiple comparisons. Predominantly produced by macrophages, IL-15 is a pro-inflammatory, immunomodulatory cytokine that stimulates T, B, and Natural Killer cells (40). Lower levels of IL-15 have been reported in the cervicovaginal environment of women with *S. haematobium* infection, with between-study differences potentially related to genital *Schistosoma* DNA detection (20). The Th2 cytokine IL-13 is thought to be an important driver of collagen deposition in the *Schistosoma* egg granuloma (41), ultimately leading to fibrosis. Indeed, IL-13 expression levels have been associated with the severity of hepatic fibrosis in *S. mansoni* infection (42). The exploratory sub-group analyses thus support previous work describing that *S. haematobium* may modulate the genital immune environment (20, 21). As a protracted Th2 response is often associated with schistosomiasis-related morbidity (43), our study findings warrant further investigation. The current study included many statistical tests, and these were allowed for using a permutation testing approach. However, we cannot exclude the possibility that some of the associations are due to chance. Thus, it may be more instructive to consider patterns in cytokine signatures (Th2, pro-inflammatory) across analyses, rather than interpreting significance testing for any one cytokine in isolation. Although strong evidence remained for some of the associations found in the exploratory analyses of FGS burden after adjusting for potential confounders and multiple comparisons, as exploratory analyses, these findings should be viewed as hypothesis generating.

Many hypotheses have been put forward regarding the mechanism of HIV-1 vulnerability in women with FGS, with evidence of both local mucosal factors and systemic immunomodulation (2, 4–6). In a South African study, women who acquired HIV-1 in the CAPRISA tenofovir gel trial had higher concentrations of the chemotactic cytokines MIP1-α (CCL-3), MIP1-β (CCL-4), IL-8 (CXCL-8), and IP-10 (CXCL-10) prior to seroconversion than women who did not seroconvert (8). In the current study, the concentrations of the aforementioned chemotactic chemokines were not higher in women with FGS compared to the FGS *negative* group. While not yet studied in *S. haematobium*, herpes simplex virus type 2 infection stimulates TNF-α production in dendritic cells, enhancing the expression of the chemokine co-receptor CCR5 and stimulating HIV-1 replication (44). Further research is needed to elucidate mechanisms for the association of FGS with HIV-1 vulnerability, and these may include schistosome-related impact on mucosal and systemic immunity, including the activation of CD4 trafficking to the genital mucosa (45), or modification in systemic (46) or cervical (20) gene expression, specifically related to the regulation of transcription, the inflammatory response or tissue fibrosis.

Cervical tissue containing *S. haematobium* eggs is more vascular (4) compared to non-egg containing tissue. Clinically, these abnormal blood vessels can be found encircling sandy patches and contact bleeding has been associated with FGS (47). Thus, FGS studies using cervicovaginal lavage are likely to be burdened by the presence of hemoglobin (48). Since HIV-1 and the presence of hemoglobin in CVL are potentially on the causal pathway between FGS and a change in cytokine or chemokine concentrations, we were unable to adjust for these possible confounding variables in a multivariable model. A sensitivity analysis removing participants with HIV-1 showed no change in the association between FGS and cytokine or chemokine concentrations. HIV-1 infection modulates the cervicovaginal immune environment in women with detectable cervicovaginal HIV-1 RNA (49) and we did not have complete data on plasma viral load in this cohort. Additionally, the analysis to remove participants with any CVL hemoglobin detection was limited by loss of power. Once participants who had any CVL hemoglobin were removed from the analysis, there was evidence of higher concentrations of IL-17A and IL-8 (CXCL-8) in participants with FGS (n=8) compared with FGS *negative* participants (n=57). This finding, however, was less robust after adjusting for multiple comparisons. Considering the potential loss of the FGS phenotype when excluding women with CVL hemoglobin and the small and likely non-representative sample size, these findings should be interpreted with caution.

Our study has a number of strengths. We are the first to describe the cervicovaginal immune environment in women with *Schistosoma* PCR-defined FGS and this study illustrates the importance of evaluating FGS burden. Defining FGS based on the detection of *Schistosoma* DNA in the female genital tract by PCR results provides a higher certainty of genital involvement and a quantitative reference standard compared to visually-diagnosed FGS or the use of urine diagnostics alone. Due to the small numbers of FGS cases, we employed a matching strategy wherein participants with FGS were frequency matched with FGS *negative* participants. To reduce the risk of selection bias, we used a random number generator to randomly select controls and matched on age group. Multiplex bead-based assays have a precedent for use in CVL (8, 11, 14, 17) and we examined a variety of soluble immune proteins including chemokines, Th1, Th2, pro-inflammatory and regulatory cytokines for a broad overview of the cervicovaginal immune environment. Another strength is that we present both crude and adjusted outcomes to facilitate comparisons in future study settings.

While our study has multiple strengths, there are also some relevant limitations. The study was conducted in a low-prevalence area and the number of FGS cases in the main and exploratory analyses is small. We selected a sub-sample of the cohort for multiplex bead-based immunoassays. This may limit the generalizability of the proportions presented for demographic variables or FGS and schistosomiasis prevalence. Secondly, we were unable to measure a number of behavioral and biological factors that also affect cervicovaginal soluble immune protein expression patterns including the presence of bacterial vaginosis (13), HSV-2 status (11), intra-vaginal cleansing practices (17), vaginal pH, menstrual cycle phase (18), body weight (50) and recent sexual contact (16). It is also undetermined how these same factors may influence *Schistosoma* DNA concentrations. Thus, we cannot exclude unmeasured and residual confounding. Additionally, due to the cross-sectional study design, we were also unable to assess the long-term impact of the cytokine expression profiles or to determine the duration of FGS infection. Though the cytokines and chemokines we measured are well-known biomarkers for inflammation and disease, it is a limitation that we did not have companion flow cytometry, biopsy, or transcriptomic data for a more detailed evaluation of cellular and histological processes. Additionally, CVL is dilute and the concentrations of the cytokines and chemokines we measured were small. This could potentially be ameliorated in future studies with the use of a menstrual cup to collect genital fluid (51).

FGS is thought to be a chronic infection of the female genital tissue, with initial infection and the development of genital lesions occurring during childhood water contact (52) that persist into adulthood, often even despite treatment with praziquantel (53). The chronicity of FGS lesions may have an impact on the cervicovaginal immune environment and a longitudinal study is needed to document the FGS immune environment in a spectrum of FGS stages before interventions can be based on our exploratory observations. Further work investigating the cervicovaginal immune environment in FGS may impact diagnostic, preventative, and therapeutic options as well as potentially providing additional information on HIV-1 vulnerability.

In conclusion, this study does not show a difference in the cervicovaginal immune environment by *Schistosoma* PCR-defined FGS status. However, two exploratory analyses suggest that there may be a relationship between higher genital *Schistosoma* DNA concentrations or multiple PCR positive genital specimens and a Th2 and pro-inflammatory modulation of the cervicovaginal immune environment, as measured by elevated cytokine concentrations. FGS may alter the female genital tract immune environment, but a larger longitudinal study in a high FGS prevalence area is needed to better define the role of FGS in HIV-1 acquisition.

## Data Availability Statement

Anonymised data from the HPTN 071 (PopART) study that support the findings of this study can be made available by the HPTN 071 (PopART) study team, subject to an application process. Further details can be obtained from AB (amaya.bustinduy@lshtm.ac.uk).

## Ethics Statement

The studies involving human participants were reviewed and approved by University of Zambia Biomedical Research Ethics Committee (reference 011-08-17), the Zambia National Health Research Authority and the London School of Hygiene and Tropical Medicine Ethics Committee (reference 14506). Permission to conduct the study was given by Livingstone District Health Office and the superintendent of Livingstone Central Hospital. The participants provided their written informed consent to participate in this study.

## Author Contributions

AS – conceptualization, data curation, formal analysis, investigation, BILHIV project administration, visualization, writing - original draft preparation. EW – conceptualization, data curation, formal analysis, supervision, visualization, writing - original draft preparation. CP– investigation, writing – review and editing. CRP – BILHIV project administration, writing – review and editing. TM – investigation, writing – review and editing. EK – investigation, writing – review and editing. MM – investigation, writing – review and editing. JM – investigation, writing – review and editing. MMM – conceptualization, resources, writing – review and editing. JC – conceptualization and resources. GD – investigation, writing – review and editing. PC – investigation, writing – review and editing. HA – resources, writing – review and editing. RH – resources, supervision, writing – review and editing. IH – resources, supervision, writing – review and editing. GM – resources, writing – review and editing. PC – investigation, writing – review and editing. LL – investigation, writing - review and editing. HH – conceptualization, supervision, writing – review and editing. SF – conceptualization, supervision, writing original draft preparation. AB – conceptualization, funding acquisition, supervision, visualization, writing - original draft preparation. All authors contributed to the article and approved the submitted version.

## Funding

AB received funding from the Wellcome Trust (Award 205954/Z/17/Z) and the Dowager Countess Eleanor Peel Trust. EW and RH received funding from MRC Grant Reference MR/K012126/1, and SF received salary from MRC Grant Reference MR/N023692/1. These awards are jointly funded by the UK Medical Research Council (MRC) and the UK Department for International Development (DFID) under the MRC/DFID Concordat agreement and is also part of the EDCTP2 program supported by the European Union. HPTN 071 (PopART) was supported by the National Institute of Allergy and Infectious Diseases (NIAID) under Cooperative Agreements UM1-AI068619, UM1-AI068617, and UM1-AI068613, with funding from the U.S. President’s Emergency Plan for AIDS Relief (PEPFAR); the International Initiative for Impact Evaluation with support from the Bill and Melinda Gates Foundation; the NIAID, the National Institute on Drug Abuse, and the National Institute of Mental Health, all part of the National Institutes of Health. EK was supported by South-Eastern Regional Health Authority, Norway project #2016055.

## Conflict of Interest

The authors declare that the research was conducted in the absence of any commercial or financial relationships that could be construed as a potential conflict of interest.

## References

[1] UNAIDS. UNAIDS Global AIDS Update, Communities at the Centre 2019 . Available at: https://www.unaids.org/en/20190716_GR2019_communities (Accessed November 2, 2020).

[2] Sturt AS Webb EL Francis SC Hayes RJ Bustinduy AL. Beyond the barrier: Female Genital Schistosomiasis as a potential risk factor for HIV-1 acquisition. Acta Trop (2020) 209:105524. 10.1016/j.actatropica.2020.105524 32416076 PMC7429987

[3] Kjetland EF Ndhlovu PD Gomo E Mduluza T Midzi N Gwanzura L. Association between genital schistosomiasis and HIV in rural Zimbabwean women. AIDS (2006) 20(4):593–600. 10.1097/01.aids.0000210614.45212.0a 16470124

[4] Jourdan PM Roald B Poggensee G Gundersen SG Kjetland EF. Increased vascularity in cervicovaginal mucosa with Schistosoma haematobium infection. PloS Negl Trop Dis (2011) 5(6):e1170. 10.1371/journal.pntd.0001170 21666790 PMC3110160

[5] Jourdan PM Holmen SD Gundersen SG Roald B Kjetland EF. HIV target cells in Schistosoma haematobium-infected female genital mucosa. Am J Trop Med Hyg (2011) 85(6):1060–4. 10.4269/ajtmh.2011.11-0135 PMC3225152 22144444

[6] Kleppa E Ramsuran V Zulu S Karlsen GH Bere A Passmore JA. Effect of female genital schistosomiasis and anti-schistosomal treatment on monocytes, CD4+ T-cells and CCR5 expression in the female genital tract. PLoS One (2014) 9(6):e98593. 10.1371/journal.pone.0098593 24896815 PMC4045760

[7] Kjetland EF Poggensee G Helling-Giese G Richter J Sjaastad A Chitsulo L. Female genital schistosomiasis due to Schistosoma haematobium. Clinical and parasitological findings in women in rural Malawi. Acta Trop (1996) 62(4):239–55. 10.1016/s0001-706x(96)00026-5 9028409

[8] Masson L Passmore JA Liebenberg LJ Werner L Baxter C Arnold KB. Genital inflammation and the risk of HIV acquisition in women. Clin Infect Dis (2015) 61(2):260–9. 10.1093/cid/civ298 PMC4565995 25900168

[9] Passmore JA Jaspan HB Masson L. Genital inflammation, immune activation and risk of sexual HIV acquisition. Curr Opin HIV AIDS (2016) 11(2):156–62. 10.1097/COH.0000000000000232 PMC6194860 26628324

[10] Osborn L Kunkel S Nabel GJ. Tumor necrosis factor alpha and interleukin 1 stimulate the human immunodeficiency virus enhancer by activation of the nuclear factor kappa B. Proc Natl Acad Sci U S A (1989) 86(7):2336–40. 10.1073/pnas.86.7.2336 PMC286907 2494664

[11] Masson L Mlisana K Little F Werner L Mkhize NN Ronacher K. Defining genital tract cytokine signatures of sexually transmitted infections and bacterial vaginosis in women at high risk of HIV infection: a cross-sectional study. Sex Transm Infect (2014) 90(8):580–7. 10.1136/sextrans-2014-051601 25107710

[12] Arnold KB Burgener A Birse K Romas L Dunphy LJ Shahabi K. Increased levels of inflammatory cytokines in the female reproductive tract are associated with altered expression of proteases, mucosal barrier proteins, and an influx of HIV-susceptible target cells. Mucosal Immunol (2016) 9(1):194–205. 10.1038/mi.2015.51 26104913

[13] Kyongo JK Crucitti T Menten J Hardy L Cools P Michiels J. Cross-Sectional Analysis of Selected Genital Tract Immunological Markers and Molecular Vaginal Microbiota in Sub-Saharan African Women, with Relevance to HIV Risk and Prevention. Clin Vaccine Immunol (2015) 22(5):526–38. 10.1128/CVI.00762-14 PMC4412937 25761460

[14] Mlisana K Naicker N Werner L Roberts L van Loggerenberg F Baxter C. Symptomatic vaginal discharge is a poor predictor of sexually transmitted infections and genital tract inflammation in high-risk women in South Africa. J Infect Dis (2012) 206(1):6–14. 10.1093/infdis/jis298 22517910 PMC3490689

[15] Gosmann C Anahtar MN Handley SA Farcasanu M Abu-Ali G Bowman BA. Lactobacillus-Deficient Cervicovaginal Bacterial Communities Are Associated with Increased HIV Acquisition in Young South African Women. Immunity (2017) 46(1):29–37. 10.1016/j.immuni.2016.12.013 28087240 PMC5270628

[16] Kyongo JK Jespers V Goovaerts O Michiels J Menten J Fichorova RN. Searching for lower female genital tract soluble and cellular biomarkers: defining levels and predictors in a cohort of healthy Caucasian women. PLoS One (2012) 7(8):e43951. 10.1371/journal.pone.0043951 22952818 PMC3432048

[17] Francis SC Hou Y Baisley K van de Wijgert J Watson-Jones D Ao TT. Immune Activation in the Female Genital Tract: Expression Profiles of Soluble Proteins in Women at High Risk for HIV Infection. PLoS One (2016) 11(1):e0143109. 10.1371/journal.pone.0143109 26814891 PMC4729472

[18] Al-Harthi L Kovacs A Coombs RW Reichelderfer PS Wright DJ Cohen MH. A menstrual cycle pattern for cytokine levels exists in HIV-positive women: implication for HIV vaginal and plasma shedding. AIDS (2001) 15(12):1535–43. 10.1097/00002030-200108170-00011 11504986

[19] Lyke KE Dabo A Sangare L Arama C Daou M Diarra I. Effects of concomitant Schistosoma haematobium infection on the serum cytokine levels elicited by acute Plasmodium falciparum malaria infection in Malian children. Infect Immun (2006) 74(10):5718–24. 10.1128/IAI.01822-05 PMC1594876 16988248

[20] Dupnik KM Lee MH Mishra P Reust MJ Colombe S Haider SR. Altered cervical mucosal gene expression and lower IL-15 levels in women with S. haematobium but not S. mansoni infection. J Infect Dis (2019) 219(11):1777–85. 10.1093/infdis/jiy742 PMC6500550 30590736

[21] Leutscher PD Pedersen M Raharisolo C Jensen JS Hoffmann S Lisse I. Increased prevalence of leukocytes and elevated cytokine levels in semen from Schistosoma haematobium-infected individuals. J Infect Dis (2005) 191(10):1639–47. 10.1086/429334 15838790

[22] Hayes RJ Donnell D Floyd S Mandla N Bwalya J Sabapathy K. Effect of Universal Testing and Treatment on HIV Incidence - HPTN 071 (PopART). N Engl J Med (2019) 381(3):207–18. 10.1056/NEJMoa1814556 PMC6587177 31314965

[23] Sturt AS Webb EL Phiri CR Mweene T Chola N van Dam GJ. Genital self-sampling compared with cervicovaginal lavage for the diagnosis of female genital schistosomiasis in Zambian women: The BILHIV study. PLoS Negl Trop Dis (2020) 14(7):e0008337. 10.1371/journal.pntd.0008337 32663222 PMC7360036

[24] World Health Organization. “Female genital schistosomiasis: a pocket atlas for clinical health-care professionals”, in: World Health Organization (2015). Available at: http://www.who.int/iris/handle/10665/180863 (Accessed November 2, 2020).

[25] Zambian Ministry of Health. Guidelines for the Etiological and Clinical Management of Sexually Transmitted Infections in Zambia. (2017) 1–48.

[26] Corstjens PL De Dood CJ Kornelis D Fat EM Wilson RA Kariuki TM. Tools for diagnosis, monitoring and screening of Schistosoma infections utilizing lateral-flow based assays and upconverting phosphor labels. Parasitology (2014) 141(14):1841–55. 10.1017/S0031182014000626 PMC4265670 24932595

[27] van Lieshout L Polderman AM Deelder AM. Immunodiagnosis of schistosomiasis by determination of the circulating antigens CAA and CCA, in particular in individuals with recent or light infections. Acta Trop (2000) 77(1):69–80. 10.1016/s0001-706x(00)00115-7 10996122

[28] Corstjens P de Dood CJ Knopp S Clements MN Ortu G Umulisa I. Circulating Anodic Antigen (CAA): A Highly Sensitive Diagnostic Biomarker to Detect Active Schistosoma Infections-Improvement and Use during SCORE. Am J Trop Med Hyg (2020) 103(1_Suppl):50–7. 10.4269/ajtmh.19-0819 PMC7351307 32400344

[29] Obeng BB Aryeetey YA de Dood CJ Amoah AS Larbi IA Deelder AM. Application of a circulating-cathodic-antigen (CCA) strip test and real-time PCR, in comparison with microscopy, for the detection of Schistosoma haematobium in urine samples from Ghana. Ann Trop Med Parasitol (2008) 102(7):625–33. 10.1179/136485908X337490 18817603

[30] Pillay P Taylor M Zulu SG Gundersen SG Verweij JJ Hoekstra P. Real-time polymerase chain reaction for detection of Schistosoma DNA in small-volume urine samples reflects focal distribution of urogenital Schistosomiasis in primary school girls in KwaZulu Natal, South Africa. Am J Trop Med Hyg (2014) 90(3):546–52. 10.4269/ajtmh.13-0406 PMC3945702 24470560

[31] Jespers V van de Wijgert J Cools P Verhelst R Verstraelen H Delany-Moretlwe S. The significance of Lactobacillus crispatus and L. vaginalis for vaginal health and the negative effect of recent sex: a cross-sectional descriptive study across groups of African women. BMC Infect Dis (2015) 15:115. 10.1186/s12879-015-0825-z 25879811 PMC4351943

[32] Jiang Y Zhang L Kong F Zhang M Lv H Liu G. MCPerm: a Monte Carlo permutation method for accurately correcting the multiple testing in a meta-analysis of genetic association studies. PLoS One (2014) 9(2):e89212. 10.1371/journal.pone.0089212 24586601 PMC3931718

[33] Allen JE Maizels RM. Diversity and dialogue in immunity to helminths. Nat Rev Immunol (2011) 11(6):375–88. 10.1038/nri2992 21610741

[34] Mutapi F Winborn G Midzi N Taylor M Mduluza T Maizels RM. Cytokine responses to Schistosoma haematobium in a Zimbabwean population: contrasting profiles for IFN-gamma, IL-4, IL-5 and IL-10 with age. BMC Infect Dis (2007) 7:139. 10.1186/1471-2334-7-139 18045464 PMC2222613

[35] Fu CL Odegaard JI Herbert DR Hsieh MH. A novel mouse model of Schistosoma haematobium egg-induced immunopathology. PLoS Pathog (2012) 8(3):e1002605. 10.1371/journal.ppat.1002605 22479181 PMC3315496

[36] van den Biggelaar AH Borrmann S Kremsner P Yazdanbakhsh M. Immune responses induced by repeated treatment do not result in protective immunity to Schistosoma haematobium: interleukin (IL)-5 and IL-10 responses. J Infect Dis (2002) 186(10):1474–82. 10.1086/344352 12404164

[37] Fallon PG Richardson EJ McKenzie GJ McKenzie AN. Schistosome infection of transgenic mice defines distinct and contrasting pathogenic roles for IL-4 and IL-13: IL-13 is a profibrotic agent. J Immunol (2000) 164(5):2585–91. 10.4049/jimmunol.164.5.2585 10679097

[38] Amiri P Locksley RM Parslow TG Sadick M Rector E Ritter D. Tumour necrosis factor alpha restores granulomas and induces parasite egg-laying in schistosome-infected SCID mice. Nature (1992) 356(6370):604–7. 10.1038/356604a0 1560843

[39] King CL Malhotra I Mungai P Wamachi A Kioko J Muchiri E. Schistosoma haematobium-induced urinary tract morbidity correlates with increased tumor necrosis factor-alpha and diminished interleukin-10 production. J Infect Dis (2001) 184(9):1176–82. 10.1086/323802 11598841

[40] Chehimi J Marshall JD Salvucci O Frank I Chehimi S Kawecki S. IL-15 enhances immune functions during HIV infection. J Immunol (1997) 158(12):5978–87. 9190952

[41] Dessein A Kouriba B Eboumbou C Dessein H Argiro L Marquet S. Interleukin-13 in the skin and interferon-gamma in the liver are key players in immune protection in human schistosomiasis. Immunol Rev (2004) 201:180–90. 10.1111/j.0105-2896.2004.00195.x 15361241

[42] Mutengo MM Mduluza T Kelly P Mwansa JCL Kwenda G Musonda P. Low IL-6, IL-10, and TNF-alpha and High IL-13 Cytokine Levels Are Associated with Severe Hepatic Fibrosis in Schistosoma mansoni Chronically Exposed Individuals. J Parasitol Res (2018) 2018:9754060. 10.1155/2018/9754060 29610679 PMC5828471

[43] Pearce EJ MacDonald AS. The immunobiology of schistosomiasis. Nat Rev Immunol (2002) 2(7):499–511. 10.1038/nri843 12094224

[44] Marsden V Donaghy H Bertram KM Harman AN Nasr N Keoshkerian E. Herpes simplex virus type 2-infected dendritic cells produce TNF-alpha, which enhances CCR5 expression and stimulates HIV production from adjacent infected cells. J Immunol (2015) 194(9):4438–45. 10.4049/jimmunol.1401706 25840914

[45] Yegorov S Joag V Galiwango RM Good SV Mpendo J Tannich E. Schistosoma mansoni treatment reduces HIV entry into cervical CD4+ T cells and induces IFN-I pathways. Nat Commun (2019) 10(1):2296. 10.1038/s41467-019-09900-9 31127086 PMC6534541

[46] Dupnik KM Reust MJ Vick KM Yao B Miyaye D Lyimo E. Gene Expression Differences in Host Response to Schistosoma haematobium Infection. Infect Immun (2019) 87(1):e00291–18. 10.1128/IAI.00291-18 PMC6300631 30323023

[47] Kjetland EF Ndhlovu PD Mduluza T Gomo E Gwanzura L Mason PR. Simple clinical manifestations of genital Schistosoma haematobium infection in rural Zimbabwean women. Am J Trop Med Hyg (2005) 72(3):311–9. ; 10.4269/ajtmh.2005.72.311 15772328

[48] Pillay P van Lieshout L Taylor M Sebitloane M Zulu SG Kleppa E. Cervical cytology as a diagnostic tool for female genital schistosomiasis: Correlation to cervical atypia and Schistosoma polymerase chain reaction. CytoJournal (2016) 13:10. 10.4103/1742-6413.180784 27168759 PMC4854169

[49] Herold BC Keller MJ Shi Q Hoover DR Carpenter CA Huber A. Plasma and mucosal HIV viral loads are associated with genital tract inflammation in HIV-infected women. J Acquir Immune Defic Syndr (2013) 63(4):485–93. 10.1097/QAI.0b013e3182961cfc PMC3706034 23591635

[50] Mave V Erlandson KM Gupte N Balagopal A Asmuth DM Campbell TB. Inflammation and Change in Body Weight With Antiretroviral Therapy Initiation in a Multinational Cohort of HIV-Infected Adults. J Infect Dis (2016) 214(1):65–72. 10.1093/infdis/jiw096 26962236 PMC4907416

[51] Archary D Liebenberg LJ Werner L Tulsi S Majola N Naicker N. Randomized Cross-Sectional Study to Compare HIV-1 Specific Antibody and Cytokine Concentrations in Female Genital Secretions Obtained by Menstrual Cup and Cervicovaginal Lavage. PLoS One (2015) 10(7):e0131906. 10.1371/journal.pone.0131906 26147923 PMC4492781

[52] Hegertun IE Sulheim Gundersen KM Kleppa E Zulu SG Gundersen SG Taylor M. S. haematobium as a common cause of genital morbidity in girls: a cross-sectional study of children in South Africa. PLoS Negl Trop Dis (2013) 7(3):e2104. 10.1371/journal.pntd.0002104 23556009 PMC3605138

[53] Kjetland EF Mduluza T Ndhlovu PD Gomo E Gwanzura L Midzi N. Genital schistosomiasis in women: a clinical 12-month in vivo study following treatment with praziquantel. Trans R Soc Trop Med Hyg (2006) 100(8):740–52. 10.1016/j.trstmh.2005.09.010 16406034

