# Supplementary material for: Cervicovaginal Immune Activation in Zambian Women With Female Genital Schistosomiasis
Source: Front Immunol. 2021 Mar 2;12:620657. doi: 10.3389/fimmu.2021.620657 (PMC7961922; doi:10.3389/fimmu.2021.620657)
Supplement: Supplementary file 1 [file DataSheet_1.docx]

**S1 Table – Crude concentrations of eleven cytokines and six chemokines in the cervicovaginal lavage of 212 Zambian women**

| **Analyte** | **% (n)**  **Above LLOQ** | **Median (IQR) ^*^**  **concentration** | **Mean (SD) ***  **concentration** | **LLOQ**  **Serum** | **LLOQ**  **CVL** |
| --- | --- | --- | --- | --- | --- |
| Eotaxin | 73.6 (156) | 4.83 (3.60 – 6.64) | 5.22 (2.46) | 4.0 | 0.98 |
| Interferon–γ | 84.0 (178) | 1.03 (0.70 – 1.67) | 2.03 (4.44) | 0.8 | 0.33 |
| IL–10 | 90.1 (191) | 1.21 (0.75 – 2.55) | 2.53 (6.00) | 1.1 | 0.33 |
| IL–13 | 32.6 (69) | 0.76 (0.49 – 1.35) | 1.10 (1.09) | 1.3 | 0.31 |
| IL–15 | 47.2 (100) | 0.75 (0.48 – 1.24) | 1.09 (1.10) | 1.2 | 0.34 |
| IL–17A | 93.4 (198) | 1.52 (0.92 – 3.04) | 4.32 (14.64) | 0.7 | 0.26 |
| IL1–α | 99.5 (211) | 50.00 (19.89 – 164.86) | 225.99 (551.30) | 9.4 | 0.97 |
| IL1–β | 93.4 (198) | 13.05 (2.88 – 72.83) | 161.49 (414.08) | 0.8 | 0.35 |
| IL–4 | 96.2 (204) | 2.87 (1.63 – 5.03) | 3.86 (3.49) | 4.5 | 0.31 |
| IL–5 | 17.0 (36) | 0.50 (0.38 – 0.72) | 0.64 (0.54) | 0.5 | 0.32 |
| IL–6 | 87.7 (186) | 4.09 (1.56 – 13.66) | 13.54 (43.36) | 0.9 | 0.32 |
| IL–8 | 100.0 (212) | 531.22 (230.01 – 1268.70) | 1034.25 (1261.70) | 0.4 | 5.66 |
| IP–10 | 100.0 (212) | 101.74 (32.66 – 276.30) | 393.23 (1143.74) | 8.6 | 1.37 |
| MCP–1 | 100.0 (212) | 27.84 (8.15 – 72.09) | 76.83 (191.26) | 1.9 | 1.03 |
| MIP1–α | 98.1 (208) | 4.27 (2.12 – 15.04) | 13.18 (27.32) | 2.9 | 0.55 |
| MIP1–β | 83.5 (177) | 2.02 (0.85 – 5.44) | 6.59 (19.29) | 3.0 | 0.28 |
| TNF–α | 41.0 (87) | 1.18 (0.53 – 3.77) | 4.99 (13.04) | 0.7 | 0.32 |

Abbreviations: IQR – interquartile range, LLOQ – lower limit of quantification, SD – standard deviation, CVL – cervicovaginal lavage

^*^ Concentrations are reported in pg/mL

This table shows the concentrations of soluble immune proteins prior to imputation for those analytes below the limit of quantification.

**S2 Table – Principal component analysis on data from log-transformed cytokines and chemokines**

|  | **Comp1** | **Comp2** | **Comp3** | **Comp4** | **Comp5** | **Comp6** | **Comp7** | **Comp8** | **Comp9** | **Comp10** | **Comp11** | **Comp12** | **Comp13** | **Comp14** | **Comp15** | **Comp16** | **Comp17** |
| --- | --- | --- | --- | --- | --- | --- | --- | --- | --- | --- | --- | --- | --- | --- | --- | --- | --- |
| Eotaxin | 0.099 | 0.597 | -0.123 | 0.295 | -0.343 | 0.423 | 0.214 | -0.098 | -0.132 | 0.266 | 0.196 | 0.011 | 0.008 | 0.099 | 0.012 | 0.201 | 0.051 |
| INF–**γ** | 0.258 | 0.154 | -0.158 | -0.234 | -0.099 | -0.119 | -0.585 | -0.149 | 0.147 | 0.364 | 0.017 | -0.159 | -0.241 | -0.095 | 0.432 | -0.048 | -0.072 |
| IL–10 | 0.295 | 0.026 | -0.021 | -0.094 | -0.232 | -0.272 | -0.170 | -0.023 | 0.166 | -0.110 | -0.197 | 0.038 | -0.023 | 0.049 | -0.453 | 0.652 | 0.181 |
| IL–13 | 0.262 | -0.020 | -0.262 | -0.334 | -0.003 | 0.091 | 0.228 | -0.153 | -0.105 | -0.285 | -0.165 | 0.200 | 0.480 | -0.066 | 0.472 | 0.207 | -0.088 |
| IL–15 | 0.268 | 0.284 | -0.205 | -0.074 | 0.202 | 0.366 | -0.254 | 0.026 | -0.340 | -0.246 | -0.074 | -0.528 | 0.063 | 0.091 | -0.330 | -0.098 | -0.224 |
| IL–17a | 0.248 | 0.225 | -0.076 | 0.261 | 0.140 | -0.178 | -0.257 | 0.583 | -0.370 | -0.132 | -0.043 | 0.402 | -0.023 | -0.086 | 0.089 | -0.089 | 0.127 |
| IL1–α | 0.206 | -0.212 | -0.336 | 0.333 | 0.430 | 0.078 | -0.044 | -0.052 | 0.349 | 0.211 | 0.387 | 0.106 | 0.244 | -0.265 | -0.135 | 0.129 | -0.043 |
| IL1–β | 0.265 | -0.284 | 0.008 | 0.299 | 0.182 | 0.064 | 0.180 | 0.102 | 0.065 | 0.093 | -0.219 | -0.287 | -0.146 | 0.466 | 0.348 | 0.114 | 0.407 |
| IL–4 | 0.260 | 0.310 | -0.075 | 0.161 | -0.151 | -0.075 | 0.102 | -0.003 | 0.571 | -0.293 | -0.264 | -0.075 | 0.105 | -0.043 | -0.087 | -0.506 | 0.050 |
| IL–5 | 0.225 | 0.014 | -0.278 | -0.437 | 0.034 | -0.083 | 0.521 | 0.373 | 0.046 | 0.132 | 0.175 | -0.085 | -0.420 | -0.118 | -0.098 | -0.037 | -0.061 |
| IL–6 | 0.246 | -0.175 | 0.367 | 0.029 | -0.076 | 0.239 | 0.098 | 0.155 | -0.041 | 0.478 | -0.492 | 0.029 | 0.148 | -0.381 | -0.091 | -0.028 | -0.169 |
| IL–8 | 0.259 | 0.089 | 0.143 | 0.247 | 0.269 | -0.157 | 0.191 | -0.541 | -0.211 | -0.190 | -0.108 | 0.119 | -0.474 | -0.218 | 0.030 | 0.037 | -0.189 |
| IP–10 | 0.163 | 0.395 | 0.369 | -0.221 | 0.420 | -0.351 | 0.107 | -0.072 | -0.060 | 0.262 | 0.132 | -0.111 | 0.360 | 0.271 | -0.093 | -0.053 | 0.019 |
| MCP–1 | 0.190 | 0.036 | 0.464 | -0.285 | 0.168 | 0.535 | -0.151 | 0.051 | 0.254 | -0.250 | 0.243 | 0.249 | -0.185 | 0.007 | 0.033 | 0.044 | 0.204 |
| MIP1–α | 0.267 | -0.188 | 0.264 | 0.196 | -0.293 | -0.126 | 0.017 | 0.188 | 0.099 | -0.118 | 0.305 | -0.010 | 0.027 | 0.298 | 0.126 | 0.071 | -0.649 |
| MIP1–β | 0.266 | -0.200 | 0.198 | 0.040 | -0.344 | -0.166 | 0.051 | -0.098 | -0.252 | -0.103 | 0.407 | -0.288 | 0.163 | -0.374 | 0.030 | -0.157 | 0.420 |
| TNF–α | 0.266 | -0.275 | -0.179 | -0.128 | -0.174 | 0.046 | -0.041 | -0.286 | -0.169 | 0.222 | 0.026 | 0.463 | -0.003 | 0.403 | -0.277 | -0.385 | 0.092 |
| POV | 0.519 | 0.087 | 0.081 | 0.062 | 0.046 | 0.037 | 0.027 | 0.024 | 0.021 | 0.018 | 0.017 | 0.015 | 0.013 | 0.011 | 0.011 | 0.007 | 0.005 |
| CPOV | 0.519 | 0.606 | 0.687 | 0.749 | 0.795 | 0.831 | 0.856 | 0.882 | 0.903 | 0.921 | 0.939 | 0.954 | 0.966 | 0.977 | 0.989 | 0.995 | 1.000 |

Abbreviations: Comp – component, CPOV – cumulative proportion of variance explained, IL–interleukin, INF –**γ** Interferon-**γ,** MCP1 – monocyte chemoattractant protein-1, MIP1-α – macrophage inflammatory protein 1–α, MIP1-β – macrophage inflammatory protein 1-β, POV – proportion of variance explained, TNF-α – Tumor necrosis factor–α

**S1** **Figure – Causal diagram for the association between FGS and a concentration change of cytokines and chemokines**


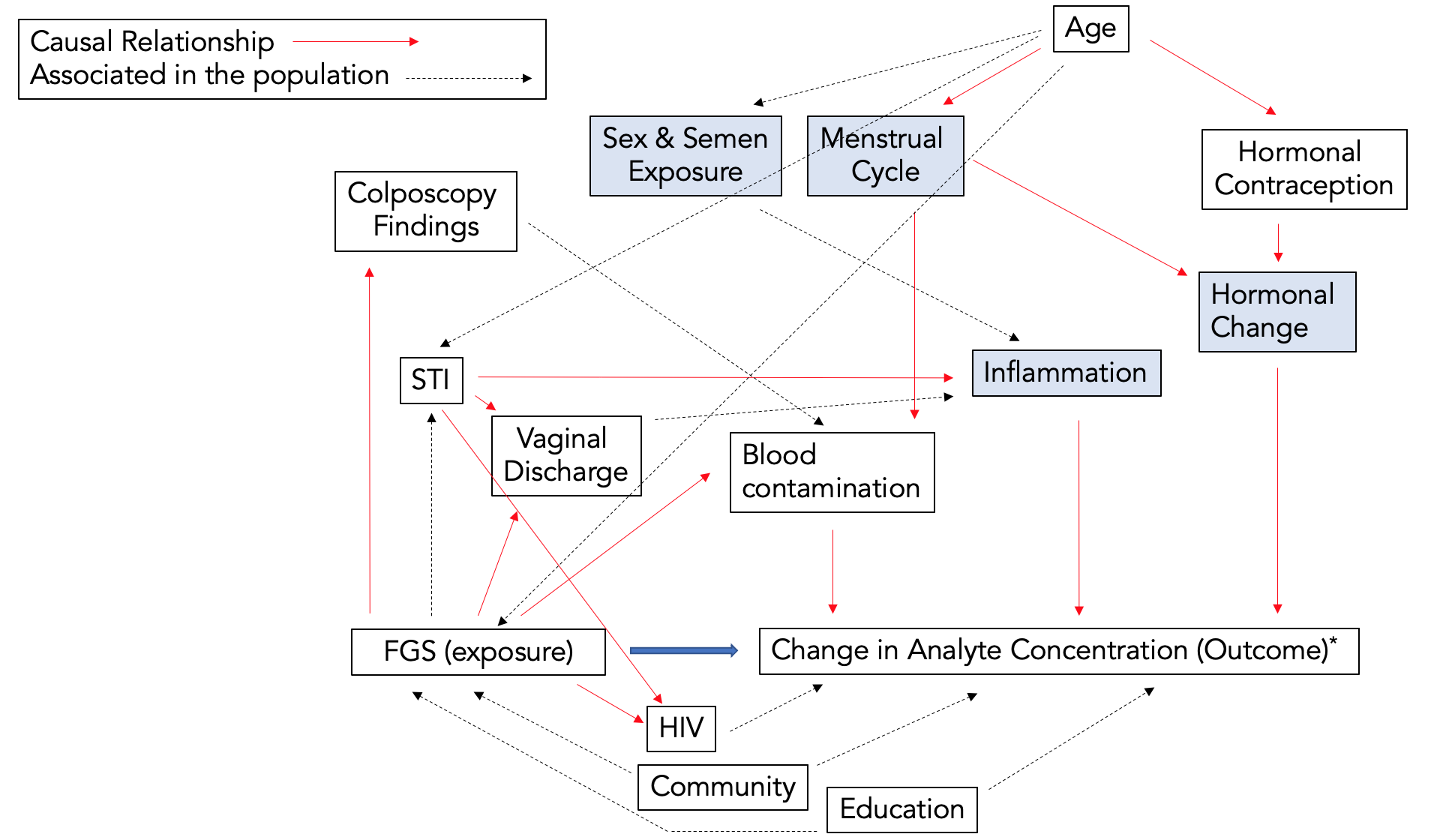


Shaded items were not measured objectively.

Adapted from Francis SC, et al. Immune Activation in the Female Genital Tract: Expression Profiles of Soluble Proteins in Women at High Risk for HIV Infection. *PloS one* (2016) 11(1):e0143109.

**S2 Figure – Spearman’s Rank Correlations by Cytokine and Chemokine Panel**


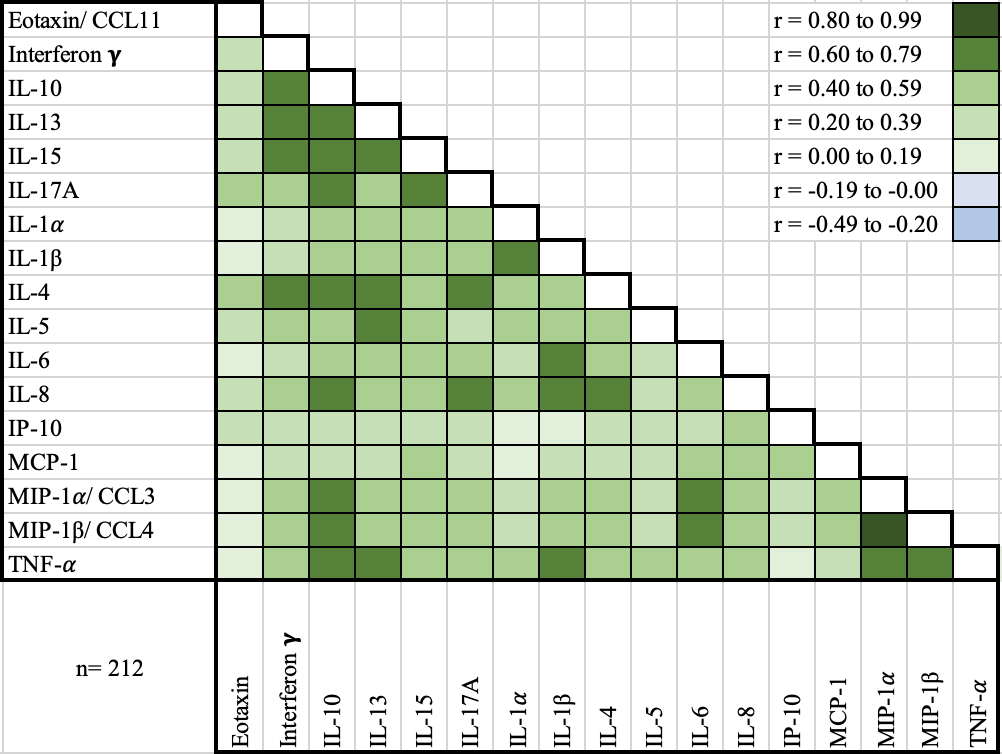


**S3 Figure – Scatterplot of the first two principal component scores by FGS Status**


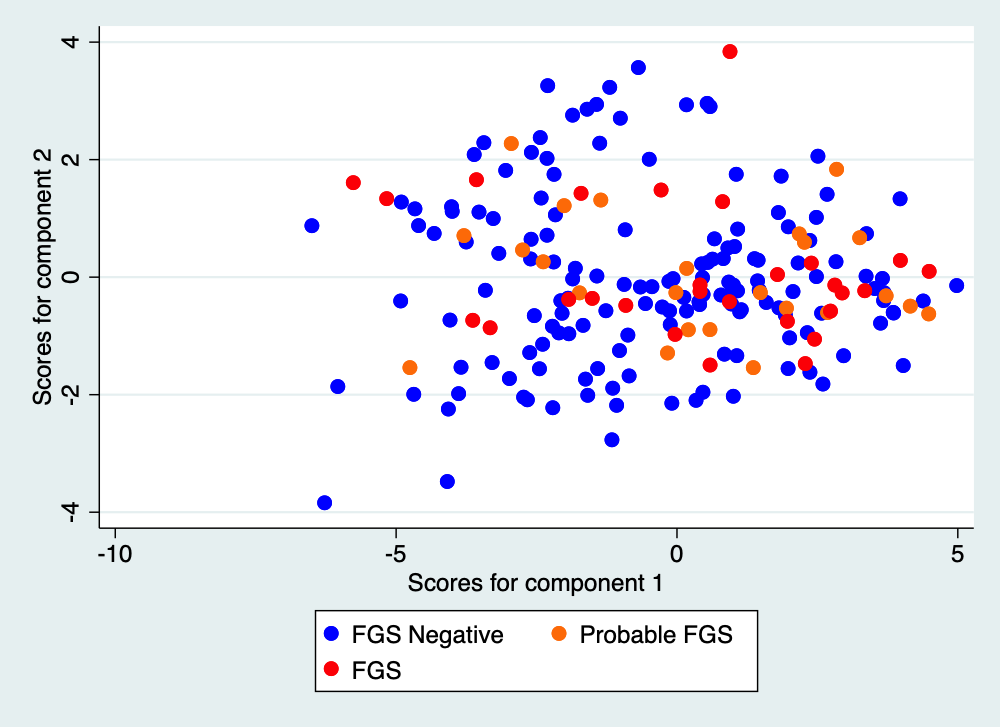


**S4 Figure – Median with interquartile range of the log-transformed crude concentrations of eleven cytokines and six chemokines in cervicovaginal lavage of participants with FGS and Probable FGS (n=53) and without female genital schistosomiasis (FGS negative: n=159)^χ^**

**^
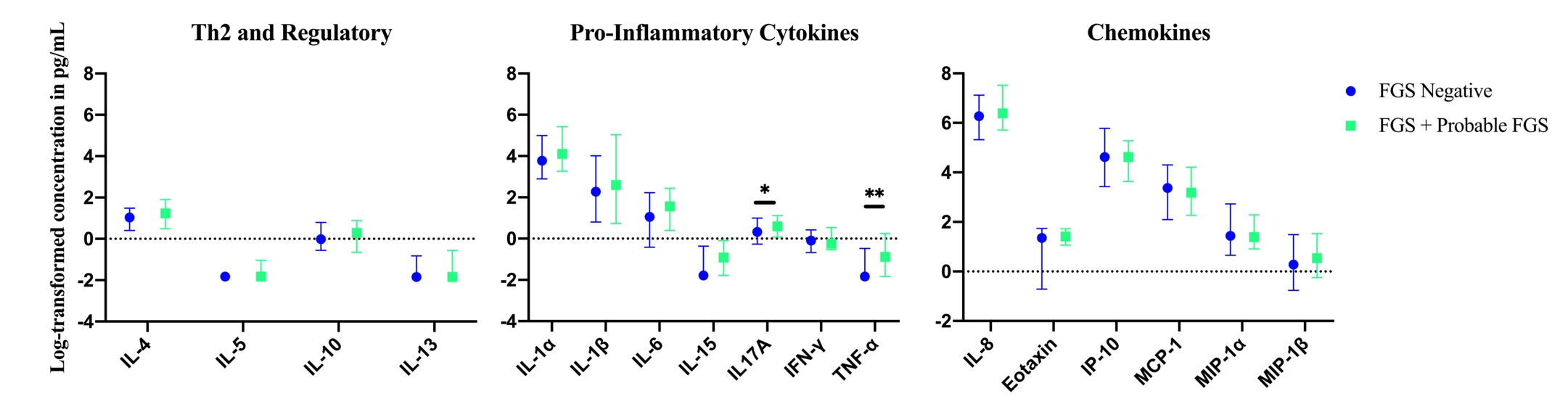
^**

**S4 Figure Caption**

**^χ^**FGS – *Schistosoma* PCR positive specimen from cervicovaginal lavage, vaginal swab or cervical swab; FGS *negative* – negative genital PCR and negative circulating anodic antigen and negative urine microscopy and negative expert-reviewed colposcopy imaging

p-value after adjustment for multiple testing with a Monte-Carlo simulation approach, p=0.09

p-value symbol legend:

*p<0.1

**p<0.05

**S5 Figure – Comparison of the concentration or presence of eleven cytokines and six chemokines in cervicovaginal lavage in participants with (FGS + Probable FGS, n=53) and without (negative FGS, n=159) female genital schistosomiasis^χ^**


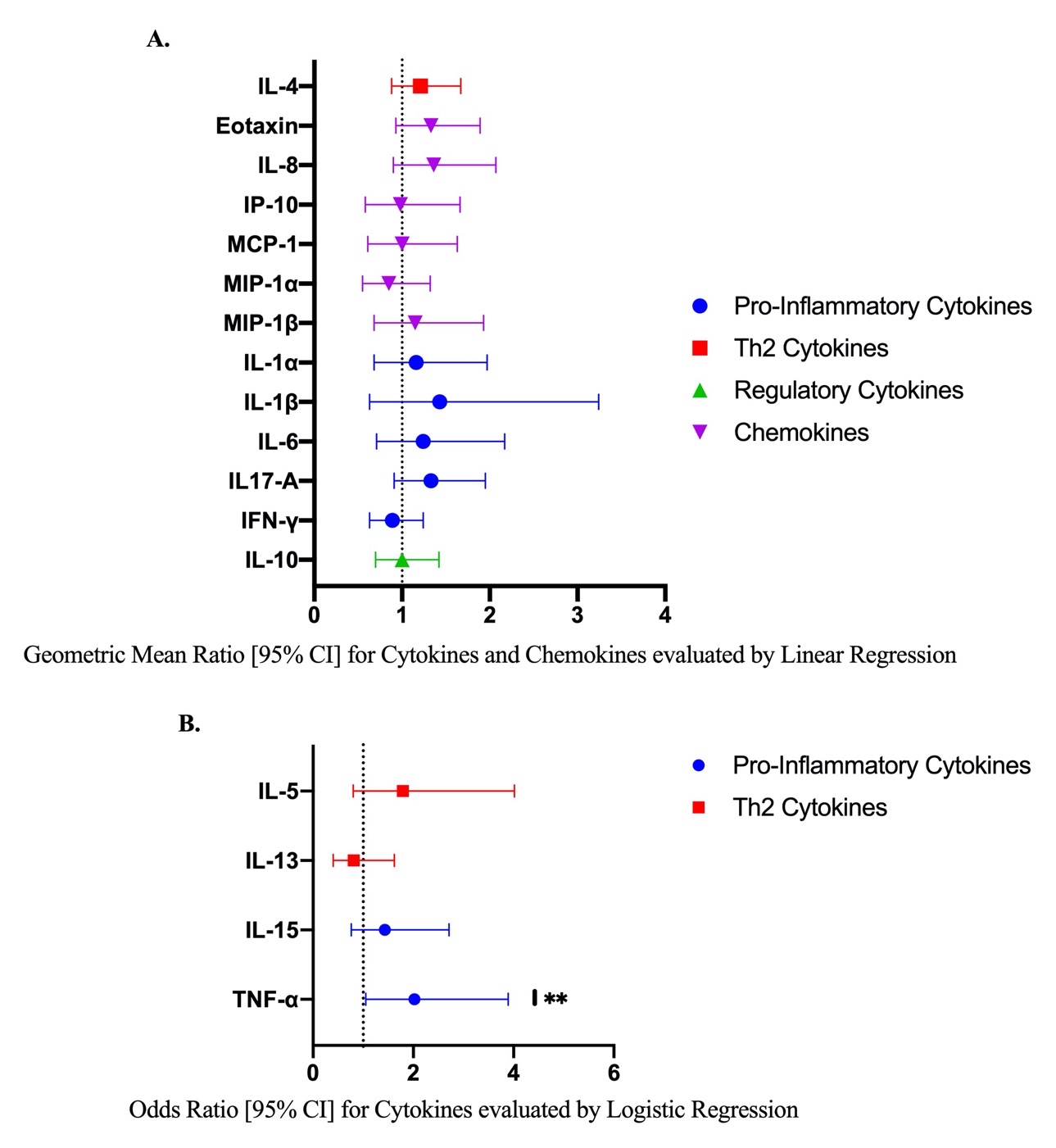


**Figure S5 Caption**

**A.** Concentrations of eotaxin, IFN-**γ**, IL-1α, IL-1β, IL-4, IL-6, IL-8, IL-10, MCP-1, MIP-1α, and MIP-1β were compared between FGS and FGS *negative* by linear regression adjusted for age, community of residence, education, presence of sexually transmitted infection and hormonal contraceptive use, with results shown as geometric mean ratios with 95% CI.

**B.** Presence/absence of IL-5, IL-13, IL-15 and TNF-α were compared by logistic regression and adjusted for age and sexually transmitted infection, with results shown as odds ratio with 95% CI.

The line at 1 indicates the value at which there is no difference between the FGS and *negative* FGS groups

p-value symbol legend:

**p<0.05

**^χ^**female genital schistosomiasis – *Schistosoma* PCR positive specimen from cervicovaginal lavage, vaginal swab or cervical swab; FGS *negative* – negative genital PCR and negative urine circulating anodic antigen and negative urine microscopy and negative expert-reviewed colposcopy imaging

**S6 Figure – Sensitivity analysis: Median with interquartile range of the log-transformed concentrations of eleven cytokines and six chemokines in cervicovaginal lavage by FGS Status (FGS: n=23, FGS negative: n=133), participants with HIV-1 excluded^χ^**


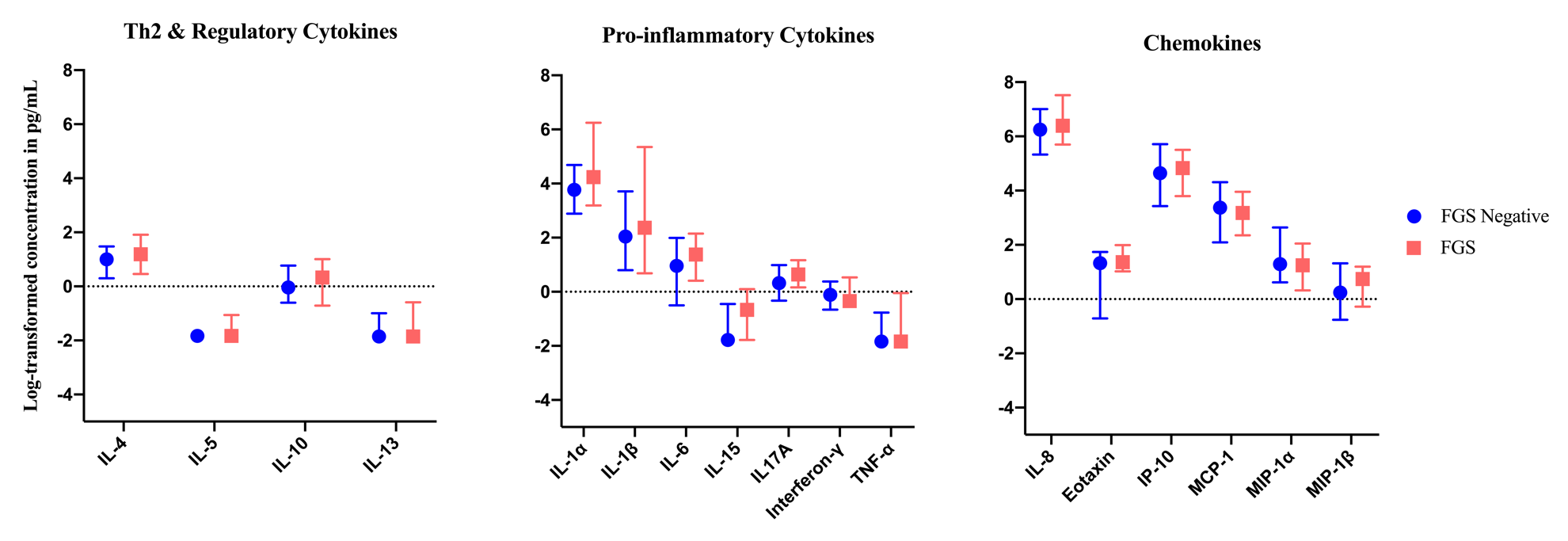


**^χ^**FGS – PCR positive specimen from cervicovaginal lavage, vaginal swab or cervical swab; FGS *negative* – negative *Schistosoma* PCR and negative circulating anodic antigen and negative urine microscopy and negative expert-reviewed colposcopy imaging

All p-values are greater than 0.1

**S7 Figure –** **Sensitivity analysis: Median with interquartile range of the log-transformed crude concentrations of eleven cytokines and six chemokines in cervicovaginal lavage by FGS Status (FGS: n=8, FGS negative: n=57), participants with hemoglobin detected in cervicovaginal lavage excluded^χ^**


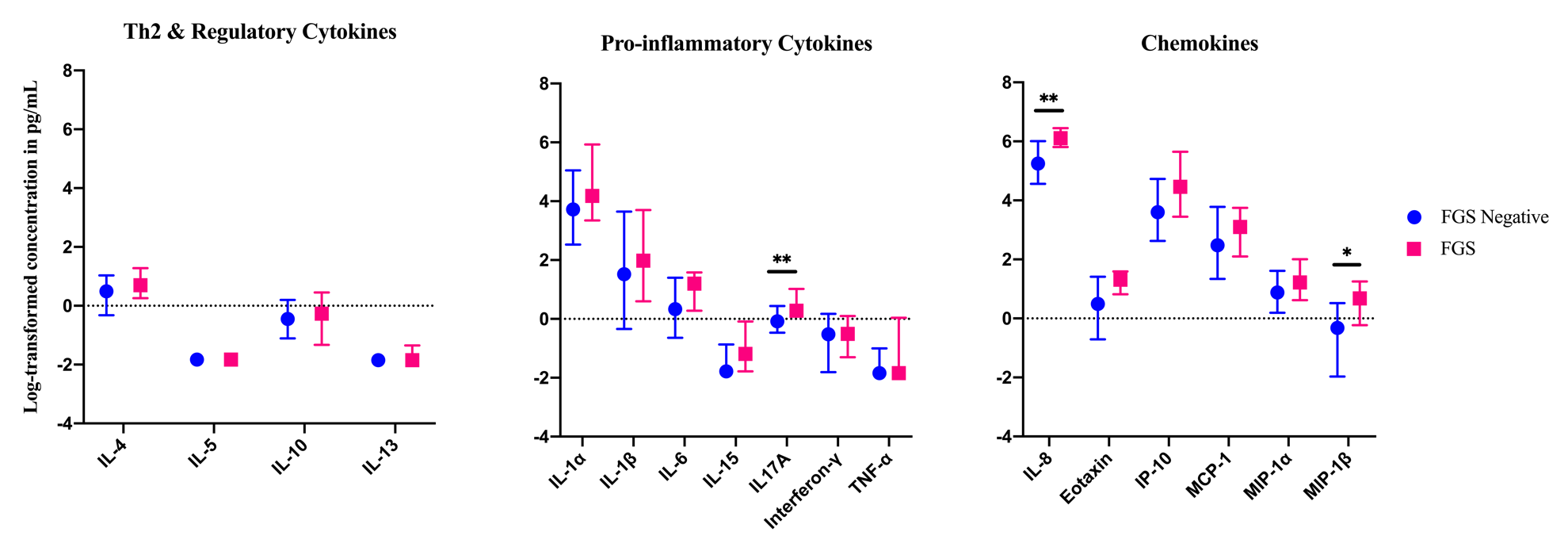


**^χ^**FGS – PCR positive specimen from cervicovaginal lavage, vaginal swab or cervical swab; FGS *negative* – negative *Schistosoma* PCR and negative circulating anodic antigen and negative urine microscopy and negative expert-reviewed colposcopy imaging

p-value after adjustment for multiple testing with a Monte-Carlo simulation approach, p=0.13

p-value symbol legend:

*p<0.1

** p<0.05
